# Regulatory T Cells As Potential Targets for HIV Cure Research

**DOI:** 10.3389/fimmu.2018.00734

**Published:** 2018-04-13

**Authors:** Adam J. Kleinman, Ranjit Sivanandham, Ivona Pandrea, Claire A. Chougnet, Cristian Apetrei

**Affiliations:** ^1^Center for Vaccine Research, University of Pittsburgh, Pittsburgh, PA, United States; ^2^Department of Microbiology and Molecular Genetics, School of Medicine, University of Pittsburgh, Pittsburgh, PA, United States; ^3^Department of Pathology, School of Medicine, University of Pittsburgh, Pittsburgh, PA, United States; ^4^Department of Infectious Diseases and Microbiology, Graduate School of Public Health, University of Pittsburgh, Pittsburgh, PA, United States; ^5^Division of Immunobiology, Department of Pediatrics, Cincinnati Children’s Hospital, Cincinnati University, Cincinnati, OH, United States

**Keywords:** regulatory T cells, FoxP3, human immunodeficiency virus, simian immunodeficiency virus, lymph node, virus eradication, cytotoxic T lymphocytes

## Abstract

T regulatory cells (Tregs) are a key component of the immune system, which maintain a delicate balance between overactive responses and immunosuppression. As such, Treg deficiencies are linked to autoimmune disorders and alter the immune control of pathogens. In HIV infection, Tregs play major roles, both beneficial and detrimental. They regulate the immune system such that inflammation and spread of virus through activated T cells is suppressed. However, suppression of immune activation also limits viral clearance and promotes reservoir formation. Tregs can be directly targeted by HIV, thereby harboring a fraction of the viral reservoir. The vital role of Tregs in the pathogenesis and control of HIV makes them a subject of interest for manipulation in the search of an HIV cure. Here, we discuss the origin and generation, homeostasis, and functions of Tregs, particularly their roles and effects in HIV infection. We also present various Treg manipulation strategies, including Treg depletion techniques and interventions that alter Treg function, which may be used in different cure strategies, to simultaneously boost HIV-specific immune responses and induce reactivation of the latent virus.

## Introduction

The human immune system walks a fine line between protection from pathogens and self-reactivity. These functions are mediated by both the innate and adaptive immune responses, such that all immune cells, from monocytes and natural killer cells to B and T lymphocytes, play integral roles in protection. Yet, a major function of the immune system, regulation, and self-tolerance, was not well understood for a long time. Gershon and Kondo in 1970 first described a population of thymus-derived lymphocytes, which were responsible for the induction of tolerance in bone marrow-derived lymphocytes (1). However, the mechanism of action and the cellular characteristics of these cells were not studied in detail until 1995, when Sakaguchi et al. reported that the CD25^hi^ CD4^+^ T cell subset has an immunoregulatory function and helps defend against the development of autoimmunity, rekindling the interest in this regulatory population (2). These cells have since been termed T regulatory cells (Tregs), and their immunosuppressive functions have been extensively investigated over the past 23 years. We now understand that Tregs are essential for proper homeostasis of the immune system and regulation of self-tolerance. While clearly playing a significant role in the pathogenesis of HIV infection, there is still debate in the field of whether Tregs are a boon or bane to fighting the virus. More recently, Tregs were reported to be involved in the HIV reservoir seeding, maintenance, and control of reactivation. In this review, we discuss Tregs and their roles during HIV infection, with emphasis on their role in viral persistence, and strategies for Treg manipulation that may have an impact for an HIV cure.

## Types of Tregs

Based on their site of differentiation, Tregs can be classified into thymic Tregs (tTregs) and peripheral Tregs (pTregs). The differences in the differentiation of tTregs and pTregs have been described in detail by Lee et al (3).

Separation based on their immunophenotypes identified numerous distinct Treg subpopulations (4) (Table 1). Treg classification through other methods, such as mass cytometry, also showed that they form a very diverse population, with up to 22 different Treg subsets being identified (5). In this section, we only focus on the key Treg subsets for which both immunophenotypes and function were well characterized.

**Table 1:** Key immunophenotypic markers/molecules and cytokines expressed by T regulatory cells (Tregs) and their function.

	Function in Tregs	Reference
**Marker/molecule**		
CD25	Receptor for IL-2, essential for Treg function and maintenance	(2)
FoxP3 (forkhead box P3)	Co-ordinates expression of various genes required for Treg activity	(6–8)
CD127 (Low)	Receptor for IL-7	(9, 10)
CTLA-4 (Cytotoxic T lymphocyte antigen-4)/CD152	Ablates CD28 costimulation by competitive binding to CD80 and CD86. Upregulation of IDO production by DCs	(11–14)
CD28	Development and maturation, activation induced Treg markers and expression of CCR6	(15, 16)
PD1 (Programmed cell death-1)	Binds to PD-L1, inhibits proliferation and effector responses of lymphocytes	(17–19)
ICOS (Inducible costimulator)/CD278	Controls expansion and maintenance of the Foxp3^+^ regulatory T cells, and IL-10 production	(20–22)
LAG-3 (Lymphocyte activation gene-3)/CD223	Plays an important role during IL-27-mediated enhanced Treg function	(23)
GITR (Glucocorticoid-induced tumor necrosis factor receptor)/CD357	Differentiation of thymic Tregs (tTregs), and expansion of both tTregs and pTregs	(24, 25)
GARP (Glycoprotein A repetitions predominant)	Present on activated Tregs; promote activation and secretion of TGF-β	(26, 27)
TNFR2 (tumor necrosis factor receptor 2)/CD120b	Promotes sustained expression of FoxP3	(28)
Helios	Highly expressed on tTregs; enhances Treg function by increasing expression of other Treg functional molecules	(29–32)
CD39	Anti-inflammatory effect by hydrolytically cleaving ATP to AMP	(33, 34)
CD73	Anti-inflammatory effect by hydrolyzing AMP cleaved by CD39 to adenosine	(32, 35)
CCR4	Expressed on effector Tregs; required for recruitment to tissue, through CCL22	(36–38)
CCR6	Regulates migration to inflammatory tissue	(39)
CCR7	Required for migration to lymph nodes; limits Treg circulation back to the thymus	(40–42)
CXCR5	Expressed on Tfr cells; required for homing to the germinal centers	(43)

**Cytokines**		
IL-10	Secreted; anti-inflammatory	(44, 45)
TGF-β	Membrane bound and secreted; suppressive; important for Treg trafficking to the gut	(46–50)
IL-35	Secreted; suppressive	(51)

Forkhead box P3 (FoxP3) is the key marker and master regulator of Tregs (6). In fact, Tregs are defined to have a CD25^hi^ FoxP3^+^ CD4^+^ phenotype. The importance of this protein was discovered when mutations in the *foxp3* gene that codes for FoxP3 were shown to cause the X-linked recessive disease, scurfy, in mice. Scurfy presents as lymphoproliferation leading to fatal autoimmunity, and mimics X-linked autoimmunity-allergic dysregulation syndrome in humans (7). Scurfy mice administered with stable Tregs, defined by FoxP3 expression and full suppressive functionality, did not develop any signs of the disease (8). FoxP3 expression can also be transiently induced following *in vitro* stimulation of nonsuppressive CD25^neg^ CD4^+^ T cells, which indicates that expression of FoxP3 alone is not responsible for the regulatory activity of T cells (52).

Thymic Tregs are defined by the expression of CD25 and FoxP3 on CD4^+^ T cells. It has been shown that CD25^hi^ CD4^+^ Treg cells develop from self-reactive thymic cells that express a T cell receptor (TCR) with high affinity for self-antigens. Differentiation occurs as an alternative mechanism to apoptosis, such that self-antigen reactivity can induce an inhibitory response instead of an autoimmune response (53). Upon TCR interaction with these peptide-major histocompatibility complex (MHC) complexes, FoxP3 is induced in the immature thymocytes (54). However, FoxP3 expression is not sufficient to create a stable Treg. Demethylation of the FoxP3 locus in the Treg-specific demethylated region (TSDR) is required to generate stable tTregs (55). In addition, CpG hypomethylation of certain loci called “Treg cell representative regions” is imprinted in Tregs, also contributing to their stability (56). Interactions between B7 molecules (CD80 and CD86), expressed on the antigen-presenting cells (APCs), and CD28, on thymocytes, are co-stimulatory and are critical to the thymic development of Tregs, as evidenced by the severe decrease in Treg numbers in mice either deficient in CD28 or treated with a blocking anti-B7 antibody (15, 57, 58). Interleukin-2 (IL-2), the central cytokine involved in Treg biology, is also essential for tTreg maturation (59).

In addition to tTregs, it has become clear that *de novo* expression of FoxP3 can occur in non-Treg CD4^+^ T cells, either *in vitro* or *in vivo*. Such induction of FoxP3 expression notably happens when naïve T cells are stimulated in the presence of transforming growth factor beta-1 (TGF-β1) (60), leading to the development of a subset of induced Tregs (iTregs). This subset has extensively been used to study the functions and characteristics of Tregs *ex vivo* (61); however, it is now recognized that these *in vitro*-induced iTregs may not accurately portray the characteristics of *in vivo*-induced, pTregs. Notably, full FoxP3 TSDR methylation does not occur in TGF-β-induced Tregs, leading to poorly suppressive and unstable Tregs (62). Conflicting reports have been published with regard to the contribution of retinoic acid (RA) to pTreg differentiation. RA from dendritic cells (DCs) was reported to be a key cofactor in generating pTregs in the gut (63, 64). However, supplementation by RA does not increase Treg frequency (65), which has cast doubt on the role really played by RA in pTreg differentiation. Interestingly, RA can phosphorylate AKT (protein kinase B) (66), thereby reducing its activity, and this pathway could be involved in pTreg differentiation, because a constitutively active AKT has been shown to impair *de novo* induction of FoxP3^+^ cells (67). Another pathway involved in pTreg induction is antigen presentation by immature DCs. Notably, it has been shown that delivering peptides in subimmunogenic forms for a prolonged period of time can result in the induction of CD4^+^CD25^+^ Tregs from naïve T cells in peripheral lymphoid organs, even in the absence of a functional thymus (68).

## Treg Homeostasis

It was thought that IL-2 is the most important Treg regulator, being required for both Treg maintenance and function (69, 70). More recently, it was shown that Tregs form two distinct populations, the CD44^lo^ CD62L^hi^ central Tregs, which actively recirculate through lymphoid organs and are sustained by paracrine IL-2, and the CD44^hi^ CD62L^lo^ CCR7^lo^ effector Tregs, which are not found in the lymphoid tissue, do not require IL-2, and are instead maintained by inducible costimulator (ICOS) (71). *In vivo*, Tregs can proliferate in response to antigens, meaning that Tregs can dynamically respond to their environment (72). It has also been shown that B7/CD28 costimulation plays a critical role in maintenance of Tregs, as shown by experiments reporting a profound decrease in Tregs in B7/CD28-deficient mice (58, 73).

## Tregs in the Lymph Nodes

L-selectin (CD62L) is thought to be crucial for the homing of Tregs to the lymph nodes (LNs). CD4^+^ CD25^+^ CD62L^+^ Tregs more potently suppress the proliferative responses of CD25^neg^ CD4^+^ T cells than CD4^+^ CD25^+^ CD62L^neg^ Tregs (74). CD62L-dependent homing induced survival and tolerance in a vascularized cardiac allograft mouse (75). It has therefore been postulated that Treg trafficking to the LNs may be dependent on CD62L (76). A CD69^neg^ CD25^+^ CD4^+^ T-cell subset from the LNs was identified to efficiently suppress CD57^+^ germinal center (GC)-Th cell-driven B cell production of immunoglobulins. These cells express CCR7 and efficiently migrate in response to CCL19, a chemokine expressed in the T cell zone of LNs. Furthermore, many of these CD69^neg^ CD25^+^ CD4^+^ T-cells populate the T cell rich zone of the LNs; however, some are present in the GCs also (77).

A particular subset of Tregs, the T follicular regulatory cells (Tfr), discovered in 2011, are Tregs that have migrated into the LN, and thus share phenotypes with Tregs, such as FoxP3, CTLA-4 (cytotoxic T-lymphocyte antigen 4), and CD25 expression (78). Additionally, they undergo differentiation and share surface markers with T follicular helper cells (Tfh), such PD-1 and ICOS, and important for their localization in the GC s, Bcl-6, CXCR5, and CXCL13 (78). This phenotype allows them to modulate B cell and Tfh cell functions in the LN follicles and acts as immune regulators of the GC responses to stimulation (43, 78–80). Currently, CTLA-4 is the only molecule demonstrated to be necessary for full suppression (81, 82); however, IL-10 and TGF-β1 are theorized to play roles in the suppressive function (83). Although a small subpopulation of mature Tfr do not express CD25 (84), depletion of Tfr by anti-CD25 mAb still enhanced humoral responses, with significantly more Ab produced (85, 86).

Soon after their migration to the LNs, Tregs form long-lasting conjugates with DCs. This prevents the DCs from interacting with CD25^neg^ CD4^+^ T helper cells (87). Using a murine mathematical model, it has been shown that after occupying the LNs, Tregs do not recirculate, whereas naïve T cells do so readily (88).

## Mechanisms of Treg Suppression

T regulatory cells produce multiple secretory cytokines that mediate their suppressive activities. The conventional dogma is that cell-to-cell contact is required for the Tregs to exert their suppressive activities (89). However, advances in cell culture capabilities have recently challenged this paradigm. A study using Treg separation from CD4^+^ T cells with a 0.45-µm permeable membrane demonstrated that, while cell-to-cell contact in the presence of IL-10 and IL-35 appears to indeed be required for Treg activation, the suppressive capabilities of Tregs are not completely mediated by cell contact. Instead, the release of inhibitory factors, such as TGF-β, IL-10, and IL-35, plays a prominent role in Treg-mediated suppression (44, 45).

Transforming growth factor beta-1 is a cytokine secreted by Tregs, which is also present on the cell surface as a membrane bound cytokine. TGF-β1 suppresses non-Treg cells through interactions with the two heterodimer TGF-β receptors, TGF-βRI and TGF-βRII (90, 91). In fact, through the use of T cell-specific *Tgfb1* deletion and subsequent Treg cotransfer experiments in *Rag1^−/−^* mice, the inhibition of Th1 differentiation and colitis was shown to be dependent upon TGF-β1 production by Tregs (46). Additional studies with TGF-β1 blockades have further supported its role as a mediator of Treg suppressive function (47, 48). TGF-β1 primarily inhibits type 1 T-helper cell (Th1) responses by blocking differentiation through the inhibition of the master regulator T-bet. However, TGF-β1 is also able to directly suppress the effector functions of CD8^+^ T cells through inhibiting cytokine and effector molecule secretion (49). Beyond direct suppression, TGF-β signaling is important for inducing Treg trafficking to the gut, where they can then modulate gut Th17 cells and gut inflammation (50).

T regulatory cells also produce IL-10, which has been shown to be important in controlling inflammation, as disruption of IL-10 production caused colitis in mice. However, unlike TGF-β1, the lack of Treg-produced IL-10 does not cause systemic immunopathology, as demonstrated through Treg-specific IL-10 deletions by Cre recombinase. On the contrary, these mice present with contained pathology to the colon, lung, and skin, indicating a tissue-specific mechanism of IL-10 immune suppression (92). Nonetheless, IL-10 has been linked to Treg activation and their effector functions (45), thus playing a critical role in immune control.

The new IL-12 family heterodimer IL-35 (Ebi3-IL-12α) is an inhibitory molecule produced by Tregs, which is required for complete suppressive functionality in mice (51). In fact, both parts of IL-35, Ebi3 and IL-12α, are necessary to support T-cell proliferation, and recombinant IL-35 was sufficient for reduction of effector T-cell proliferation (51). Tregs are capable of inducing differentiation of naïve T cells to “iT(R)35” cells (93) through IL-10 and IL-35. These iT(R)35 cells have impressive suppressive capabilities originating from substantially increased IL-35 production, while they lack FoxP3 and do not produce TGF-β or IL10, making them a population distinct from tTregs (93). However, other studies questioned the importance of IL-35 and demonstrated that IL-35 is not constitutively expressed on human Tregs, while being shown to be produced by effector T cells (94).

On the cell surface, Tregs constitutively express CTLA-4, an inhibitory receptor that ablates CD28 costimulation by competitive binding of the B7-1 and B7-2 ligands (CD80 and CD86) on APCs (11, 12). Additionally, CTLA-4 also acts through upregulation of indoleamine 2,3-dioxygenase (IDO) production by DCs, inhibiting T cell expansion (95). The importance of this protein is clearly demonstrated by the observation that mice deficient in CTLA-4 die within 2–3 weeks from major organ lymphocytic infiltration and destruction, resulting from uncontrolled lymphocyte proliferation (13). A similar fatal autoimmune disease occurs if CTLA-4 is deleted from Tregs using Cre/lox with the FoxP3 promoter, due to loss of Treg suppressive function, particularly, lack of Treg-mediated DC CD80 and CD86 expression (14). Meanwhile, CTLA-4 blockade can induce autoimmune disease (96). Further support for the suppressive function of CTLA-4 through B7-1/B7-2 was obtained by demonstrating that reversal of the lymphoproliferative phenotype occurs after the administration of the CTLA4Ig, which mimics the ablation of CD28 costimulation by CTLA-4 (97). Imaging of conventional CD4^+^ T cells, DCs, and Tregs in the LNs showed that CTLA-4 blockade increases the amount of CD4^+^ T cell-DC interactions and T-cell activation through ablation of suppressive interactions of both B7-1 and B7-1 on DCs (98). However, CTLA-4 does not act exclusively through Tregs, being also expressed on conventional T cells, where inhibitory function can occur in *cis* by both the previously stated mechanism, as well as by signaling through the cytoplasmic region (99). CTLA-4 is also an important contributor to Treg survival, as the anti-CTLA-4 mAb Ipilimumab was found to have an additional function of targeting Tregs for death by CD16^+^ nonclassical macrophages through antibody-dependent cell-mediated cytotoxicity (ADCC) (100).

An indirect Treg suppression mechanism is through consumption of IL-2. CD4^+^ CD25^+^ Tregs are able to bind IL-2, preventing non-Tregs from binding and thus inhibiting activation (101), while simultaneously depriving them of the necessary prosurvival signals to prevent apoptosis (102). This competition serves an additional function of enhancing Treg responses by priming them for IL-10 production after TCR stimulation (103, 104).

Extracellular adenosine triphosphate (ATP) is a known inflammatory signal that acts through the P2 purinergic receptors and is released from cells, which have a high intracellular concentration of ATP, during tissue damage [reviewed in Ref. (105)]. Tregs suppress the inflammatory responses to ATP through directly limiting the amount of extracellular ATP by hydrolysis of ATP to adenosine monophosphate (AMP) by CD39, which is highly expressed on the surface of FoxP3^+^ Tregs (33) and is further upregulated during inflammation (106). Indeed, CD39 plays an important role during HIV infection, as suggested by the observations that CD39^+^ Treg cells are inversely correlated with CD4^+^ T cell counts (107) and polymorphisms that cause decreased expression of CD39 correlate with slower disease progression (107) and decreased suppression of effector T cells (106). Following hydrolysis, AMP is hydrolyzed to adenosine by CD73 on the surface of Tregs (108, 109), which is then shed from the plasma membrane (110). This further increases the suppressive nature of Tregs as adenosine is an anti-inflammatory molecule. *In vitro* experiments demonstrated that adenosine directly inhibits T cell activation and proliferation through binding to the receptor A_2a_, preventing TCR-mediated IL-2R (111) and IFN-γ (112) expression. Adenosine further inhibits the Th1 response by decreasing TNF-α and IL-12 production by myeloid dendritic cells (mDCs) while simultaneously increasing IL-10 (113). Additionally, adenosine inhibits IFN-γ and IL-2 production of CD4^+^ and CD8^+^ T cells and is inversely correlated to gut inflammation and damage during SIV infection (34). Tregs also suppress T cells through cyclic AMP (cAMP). The binding of adenosine to receptors A_2A_ and A_2B_ induces adenylate cyclases, increasing the production of intracellular cAMP and suppressing the immune activation [reviewed in Ref. (114)]. Using gap junction inhibitors and cAMP antagonists, it was shown that Tregs transfer cAMP through gap junctions to suppress non-Tregs (115–117).

## Role of Tregs in HIV/SIV Infection

### Changes in Treg Frequency Throughout HIV/SIV Infection

Treg suppression of the cell-mediated immune response occurs early during the acute HIV/SIV infection, as reported in SIV-infected Rhesus macaques (RMs) (118). In fact, in HIV-infected individuals, the relative frequency of Tregs correlates with the viral load levels and disease progression (119–123), while being inversely correlated with the SIV-specific cytotoxic T lymphocyte (CTL) responses (118). Of note, CD4^+^ T cell depletion characteristic to acute HIV/SIV infection partially spares Tregs, as suggested by the observation that, in spite of the decrease in their absolute counts, Treg frequency increases during the acute HIV infection (121, 123–125). In HIV-infected subjects receiving ART, Treg frequency is partially restored (120, 126). Interestingly, elite controllers have higher Treg absolute counts, yet lower frequencies, in the peripheral blood and rectal mucosa compared to progressors (119, 127). The mechanism by which Tregs are spared relative to other CD4^+^ cell subsets during HIV infection remains unclear. In the gut mucosa of SIV-infected RMs, non-Tregs were shown to have significantly higher apoptotic gene expression than Tregs, of which some were apoptotic genes associated with HIV production, supporting the concept that Tregs are relatively resistant to cell death mediated by SIV infection (128). This relative Treg resistance to HIV/SIV infection is further supported by the observations that the number of infected Tregs is similar to CD4^+^ CD25^neg^ cells (129) and that HIV gp120 binding to CD4 enhances Treg survival (122, 130).

Alternatively, the increases in Treg frequency may also be explained by increased conversion of peripheral conventional CD4^+^ T cells to a pTreg phenotype. *Ex vivo* work performed with plasmacytoid dendritic cells (pDCs) and conventional CD4^+^ T cells from HIV-infected individuals demonstrated enhanced induction of Treg differentiation when pDCs were stimulated with HIV (131). Similarly, tissue mDCs from SIV-infected NHPs were more efficient at converting non-Tregs to Tregs (132). Increases in the levels of TGF-β characteristic to pathogenic SIV infection of RMs (133) further substantiate the theory of increased pTreg conversion during HIV/SIV infection. This finding is important, because in progressors, Treg suppressive capacity is maintained throughout infection (134), with enhanced function in the LNs, where there is a HIV/SIV reservoir of importance, compared to the peripheral blood (135–137).

In the LNs, Tfr contribute to the impairment of Tfh function (138, 139). Tfh expand during HIV infection (140–142). Their increase is associated with B cell dysfunction (143), as documented by hypergammaglobulinemia, increased GC B cells, and decreased memory B cells (140–142, 144), a likely consequence of hyperactivation through chronic antigenic stimulation (144) and increased cytokine production (141, 142). During HIV infection, the Tfh/Tfr ratio increases and is associated with impaired somatic hypermutation and affinity maturation. These functions can be restored upon Tfr reconstitution (145). While the frequency of Tfr relative to total CD4^+^ T cells increases during chronic SIV infection, the Tfr fraction of Tfh is decreased during both acute and chronic stages. Loss of Tfh proliferation control by Tfr during HIV/SIV infection has been examined as a possible explanation for Tfh expansion and may help explain the hyperactivation in the B cell follicles (146, 147). Other studies, however, reported opposite findings, showing that the Tfr/Tfh ratio increases postinfection and through expansion of the regulatory phenotype (139). These data are consistent with Tfh impairment, notably downregulation of ICOS and decreased expression of IL-21 and IL-4 (139), implicating Tfh inhibition in the aberrant humoral response. Thus, whether therapeutic targeting of Tfr in HIV/SIV infection is beneficial or detrimental is still up for debate, although their permissiveness to infection enhances the beneficial aspects of targeting Tfr (148).

### Treg Suppression in HIV Infection

**Figure 1:**
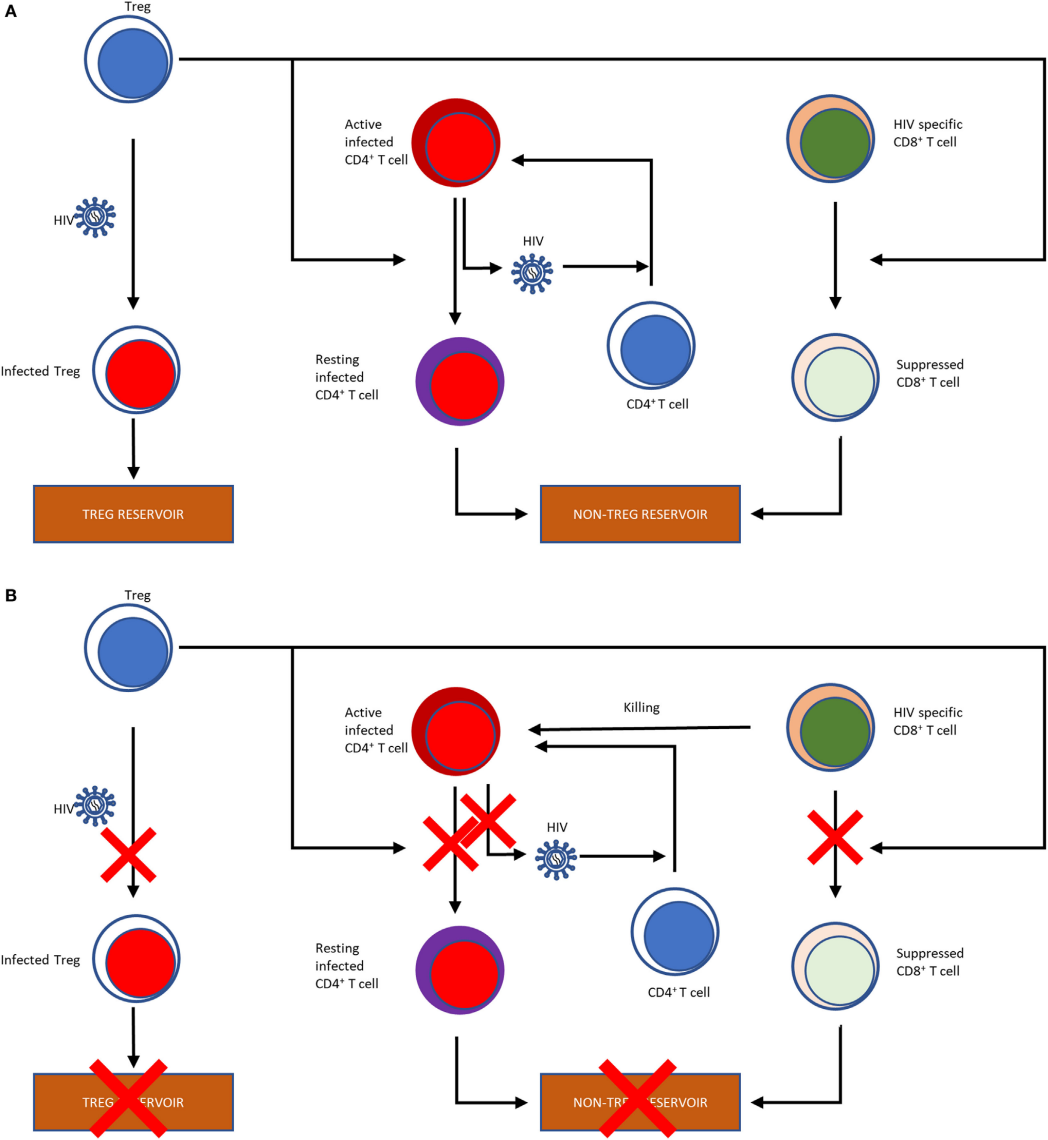
Flow chart illustrating the effects of Tregs on the HIV reservoir **(A).** Tregs can be infected with HIV, thereby contributing to the HIV reservoir. Tregs reverse the activation status of the HIV-infected T cells into resting T cells, further contributing to the reservoir formation. Finally, by suppressing the HIV-specific CD8^+^ T cells, which would otherwise kill infected cells, Tregs also shape the non-Treg reservoir. Potential effects of Treg depletion on the HIV reservoir **(B)**. Treg depletion obviously result in a reduction of the Treg reservoir through direct killing. Treg depletion also abolish their suppressive effects of the T cells, which may reverse their resting status, become activated and produce and release the virus. Finally, reversion of the suppressive effect of Tregs on HIV-specific CD8^+^ T cells has the potential to boost their anti-SIV activity, which can also curtail the reservoir.

T regulatory cells are considered both beneficial and detrimental during acute HIV infection (Figure 1). Increased immune activation is a hallmark of HIV infection, and Tregs have been shown to control the activation status of HIV-infected CD4^+^ T cells (118, 149–152). In the nonpathogenic SIV infection of African green monkeys, an increase in Tregs occurs early during the acute infection, as early as 24 h postinfection, concomitant with significant increases in TGF-β and IL-10 (153). In contrast, during the acute pathogenic SIV infection of RMs, there is only modest TGF-β induction and delayed increases in IL-10 (153). Additional support for the role of Tregs in acute infection was obtained by Cecchinato et al. who showed that CTLA-4 blockade early during the acute infection was detrimental to RMs, resulting in increased viral replication and decreased responsiveness to ART (154). These results may be due to essentially “adding fuel to the fire” by increasing immune activation and consequently expanding the target pool for SIV, although some of this may be due to non-Treg effects. *In vitro* studies have shown that, in addition to limiting the amount of susceptible cells, Tregs can limit the infection of conventional CD4^+^ T cells through DC-CD4^+^ T cell immunological synapses (117). Therefore, Tregs can help in preventing the deleterious pathogenic consequences of HIV/SIV infections by controlling the immune activation status of virus producing cells by shifting them into resting state. This thereby suppresses viral production, and prevents the spread of infection. The corollary of this paradigm is, however, that, by pushing infected T cells into a resting state, Tregs are promoting the generation of the latent HIV/SIV reservoir, the seeding of which starts as early as 3 days post-infection (155), which represents the ultimate obstacle for HIV cure research.

Meanwhile, Tregs also suppress the HIV-specific CD8^+^ T cell responses (156), their frequency being inversely correlated with the SIV-specific CTL response (118) and T-cell activation (125). The disruption of the cell-mediated immune response against HIV by Treg-mediated suppression of CD8^+^ T cytotoxic lymphocytes is therefore inhibiting viral clearance in infected individuals, likely leading to an increased viral replication. In fact, in controllers with the protective HLA-B*27 and HLA-B*57 alleles, the HLA-B*27 and HLA-B*57-restricted CD8^+^ T cells are not suppressed by Tregs, whereas the CD8^+^ T cells with nonprotective HLA alleles are highly suppressed *ex vivo*, substantiating the role CD8^+^ T cells in controlling virus (157). Jiang et al. infected humanized mice with HIV following Treg depletion and compared them with those that were not depleted of Tregs. They found that the Treg-depleted mice had lower levels of infection, as measured by peak viral loads and p24 intracellular staining in plasma and lymphoid tissues, such as spleen and mesenteric LNs (158), supporting a negative effect of Tregs on disease.

Of note, conflicting data were published with regard to the effect of Treg infection with HIV on their suppressive capabilities. Purified bulk Treg populations from HIV-infected individuals retain their suppressive activity (125, 149, 159). *In vitro*, HIV infection of Tregs has been reported to either have no effect on Treg functionality (160) or result in loss of functionality (161). However, when functionality was assessed on a per-cell basis, infected Tregs had a lower suppressive capacity and corresponding decreases in genes relating to suppressive function and increases in inhibitory genes compared to noninfected Tregs (162), and this altered suppressive capacity may further contribute to the generalized immune activation in chronic infection.

## Tregs as an HIV/SIV Reservoir

Suppression of the immune responses is not the only way that Tregs contribute to HIV disease. Treg infection with both SIV (118, 128) and HIV (160) occurs both *in vitro* (163) and *in vivo*. Indeed, when naïve T cells were transduced to express FoxP3, their susceptibility to HIV infection increased, and they produced infectious virus at levels comparable to memory T cells (163). In SIV-infected macaques, the fraction of mucosal Tregs containing SIV DNA is higher than that of the non-Tregs, but Tregs harbor less SIV RNA, which was interpreted as an indication that they are less susceptible to productive infection (128). Additionally, mucosal Tregs have a better survival rate than the non-Tregs, supporting increased infection rates without active replication (128). Similarly, in HIV-infected individuals, a larger proportion of Tregs contains HIV DNA than the non-Tregs and importantly, treatment with the histone deacetylase inhibitor (HDACi) valproic acid was able to reverse latency in resting Tregs from more patients than treatment of non-Tregs (164). Yet, when the comparisons are refined by comparing Tregs (CD25^+^ CD127^neg^) to effector memory T cells (TEM) (CD25^neg^ CD127^+^), the difference in the levels of integrated HIV DNA between the two cellular populations was no longer significant (160), an unsurprising, but notable result due to the inclusion of naïve T cells in the non-Treg group. Interestingly, when comparing the *in vitro* susceptibility to infection, Tregs were reported to be more susceptible to infection by CXCR4-tropic strains, while TEM were more susceptible to CCR5-tropic strains (160). Further substantiating Tregs as an important reservoir, replication competent virus has been reactivated from Tregs isolated from HIV-infected individuals on long-term ART (164–166).

As such, due to the increase in Treg frequency during HIV/SIV infection (118–126), a larger proportion of Tregs containing HIV/SIV DNA than non-Tregs (128, 164), better survival from SIV infection (128), and decreased suppressive activity of infected Tregs (161, 162), Tregs appear to be an important HIV reservoir. Together with their major role in shaping the viral reservoir, these data point to a key role for Tregs as targets in cure research strategies.

## Targeting Tregs as a Cure Research Strategy

The suppressive function of Tregs during HIV infection has opened the forum to assess the benefit of manipulating Tregs for the HIV-infected subjects. However, this is not without its issues, as Tregs are also beneficial in some ways, particularly in suppressing general immune activation. The major problem with targeting Tregs is that the most typical marker for Tregs, the FoxP3 molecule is intracellular and, as such, it cannot be directly targeted *in vivo*. Multiple other targets have however been considered for *in vivo* Treg depletion strategies (summarized in Table 2).

**Table 2:** Strategies for targeting T regulatory cells (Tregs) and/or their function.

Impact on Tregs	Target	Drug	Rationale	Expected Treg depletion/blockade	Reference	Potential complications
Depletion	CD25	IL-2 immunotoxin	Treg targeting through attachment to CD25, the receptor for IL-2Treg killing through eEF-2 (Eukaryotic elongation factor 2) ribosylation by diphtheria toxin	Up to 75% depletion of circulating TregsUp to 40% depletion of the lymph node (LN) Tregs	(158, 167, 168)	Autoimmunity, toxicity
			
		Daclizumab	Binds to CD25, preventing IL-2 binding and action. Il-2 is required for maintenance of Treg counts and function	Up to 50% depletion of circulating Tregs	(169–172)		
		
	CCR4	CCR4 immunotoxin	Targets Tregs by attaching to CCR4 (effector Treg marker) Treg killing through eEF-2 ribosylation by diphtheria toxin	Up to 40% depletion of circulating Tregs9–22% depletion of the LN Tregs	(173, 174)		
			
		Mogalizumab	Targeting Tregs by attaching to CCR4 and causing antibody-dependent, cell-mediated cytotoxicity	Up to 80% depletion of circulating Tregs	(175, 176)	
		
		Cyclophosphamide	Treg depletion through DNA double strand breaks and decreased DNA repair. Treg sensitivity is due to decreased production of glutathione (required for detoxification of Cy active metabolites)	Up to 50% depletion of circulating TregsIncreased CD8^+^ T cell and NK cell activation	(177, 178)	

Functional blockade	CTLA-4	Ipilimumab	Binds to CTLA-4 on Tregs, blocking it from inhibiting lymphocytes	Up to 75% decrease of circulating Tregs	(100, 154, 179, 180)	Autoimmunity, toxicity
	
	IDO	1-methyl-d-tryptophan	Inhibits IDO, blocking suppressive function	Increased expression of IFN-γ by the lymphocytes in the LNs, decreased plasma viral loads	(181, 182)	Autoimmunity

### Targeting CD25 for Treg Depletion

Two drugs targeting Treg through their constitutive expression of CD25, Daclizumab and Ontak, have been used to deplete them (183, 184). Both compounds bind CD25, but act differently. For Denileukin difitox (ONTAK), which is IL-2 coupled with diphtheria toxin, the IL-2 identifies and binds the CD25^+^ cells, allowing the diphtheria toxin to enter the cell and cause cell death by ADP-ribosylating host eEF-2 and preventing protein synthesis (185). Ontak has been used to treat CD25^+^ cutaneous T cell lymphoma (186). Ontak has also been tested with relatively positive results in peripheral T-cell lymphoma, metastatic renal cell carcinoma, and unresectable stage IV melanoma (187–189). Other studies of Ontak administration to melanoma patients, together with a DC vaccine reported no peripheral Treg depletion; yet, this result may be due to the use of a very low dose (190). In the same study, *in vitro* assessments showed that, while the internalization of Ontak was observable in activated Tregs even at low concentrations, Ontak internalization in resting Tregs only occurred at very high concentrations (190). This may be a potential barrier to the use of Ontak to target the resting reservoir.

The second component, Daclizumab, is a monoclonal antibody to CD25, which prevents the interaction of IL-2 with its receptor. As such, Daclizumab may be used for Treg depletion, as IL-2 is essential for Treg development, maintenance, and function, as discussed above. It has been approved for the treatment of relapsing forms of multiple sclerosis (169, 170) and in adult T cell leukemia to induce remission (191). Daclizumab was also used in radio-immunotherapeutic approaches, after linking it with ^90^Y, and its administration extended the length of remission in patients with adult T cell leukemia (171). This conjugated Daclizumab was also tested in other CD25^+^ malignancies, with promising results, especially for patients with Hodgkin’s disease (192). Finally, for a short time, it was used to prevent acute rejection in patients with kidney transplants (193).

In the context of HIV infection, these compounds look promising, with regards to reservoir control. Ontak administration to DKO-hu HSC mice, followed by infection with HIV-R3A, reduced the levels of Tregs in blood, spleen, and mesenteric LN and increased the expression of HLA-DR, a marker of immune activation, on CD4^+^ and CD8^+^ T cells. It nevertheless resulted in lower levels of HIV-1 present in the plasma and the lymphoid organs, during the acute stage of infection (158). Furthermore, Ontak administration to humanized NRG mice infected with HIV-1 and completely virologically suppressed by ART, resulted in viral reactivation in spleen and bone marrow. Cell-associated viral DNA levels did not change, indicating that the virions relapsed from the reservoir. The mice were maintained on ART, which prevented the reactivated virus to reinfect cells and, after virus control was achieved post-Ontak administration, the levels of cell-associated viral DNA were significantly decreased in the lymphoid tissue as compared to controls, with no significant change in total CD4^+^ T cells in the spleen and bone marrow (194).

Ontak administration to chronically SIVsab-infected African green monkeys, resulted in a significant Treg depletion and induced significant CD4^+^ and CD8^+^ T cell activation (167). Finally, Ontak administration to SIVsab-infected RMs, a model of spontaneous complete control of HIV infection (195, 196), resulted in the depletion of 75–85% of the peripheral Tregs, an 8- to 10-fold increase in immune activation of the peripheral CD4^+^ and CD8^+^ T cells and a boost of SIV-specific T cells (168). Furthermore, a relatively robust virus reactivation was observed, with plasma viral loads reaching up to 10^3^ viral RNA copies/mL (from below 5 copies/mL before treatment).

These results suggested that Treg depletion is a plausible strategy for reducing the HIV reservoir in circulation and lymphoid tissues, while boosting effective cell-mediated immune responses (168). Ontak was discontinued for clinical use due to the production issues related to difficulties in the purification from the bacterial expression system. Daclizumab has also been discontinued. However, a new bivalent IL-2 immunotoxin was developed that showed increased potency when compared to the Ontak-like monovalent version (197). When it was used in human CD25^+^ HUT102/6TG tumor-bearing NSG mouse model, this bivalent immunotoxin was shown to significantly prolong survival of the mice in a dose-dependent manner (198).

### Targeting CCR4 for Treg Depletion

T regulatory cells (Tregs) express a high level of CCR4 (199–201), which is the receptor for CC chemokines (MIP-1, RANTES, TARC, and MCP-1) and has been shown to be a coreceptor for HIV-1 (202). Wang et al. developed a diphtheria-toxin based anti-human CCR4 immunotoxin, which effectively binds to and cause protein synthesis inhibition in target cells. It prolongs the survival of tumor-bearing NOD/SCID IL-2 receptor γ−/− (NSG) mice injected with human CCR4^+^ acute lymphoblastic leukemia cells, indicating the efficacy of this drug (173). When the drug was tested in NHPs, it depleted ~80% of CCR4^+^ FoxP3^+^ and 40% of FoxP3^+^ CD4^+^ T cells in the peripheral blood. In the LNs, although there was a decrease of ~90% of CCR4^+^ FoxP3^+^ Tregs, overall FoxP3^+^ CD4^+^ T cells were decreased by only 9–22% (174). The anti-CCR4 monoclonal antibody, Mogamulizumab, also shows promise in treatment of peripheral T-cell lymphoma and cutaneous T-cell lymphomas like mycosis fungoides and Sezary syndrome, by depleting CCR4^+^ malignant cells and CCR4^+^ Tregs (175, 176).

### Cyclophosphamide

Cyclophosphamide (Cy) is a well-established chemotherapeutic agent, which is widely used for the treatment of leukemias and lymphomas. In high doses, Cy acts as a nonselective cytoreductive agent, which directed its uses as part of a preparation regimen for allogeneic stem-cell transplantation (203) and in treatments for systemic lupus erythematosus (SLE) (204–206). In low, metronomic dosages, Cy retains its antitumor capabilities, with reduced side effects and improved clinical responses (207). In mice, low-dose Cy administration selectively and significantly depleted and reduced the functionality of Tregs (208, 209). *In vitro*, CTLs and T helper cells are more resistant to Cy cytotoxicity than Tregs (210). Treg selectivity has been attributed to decreased DNA repair, as demonstrated by the increased and sustained DNA intercross-linking, as well as increased and sustained phosphorylated histone 2AX. A different mechanism for sensitivity, decreased production of glutathione, a detoxifier for Cy and its active metabolites, was also evoked. Indeed, Tregs have decreased ATP levels, which abrogate glutathione production, thereby inducing hypersensitivity to Cy (211). Interestingly, CCR2^+^ Tregs are preferentially depleted in mice over CCR2^neg^ Tregs. An analysis of the cell cycles demonstrated increased proliferation and activation in CCR2^+^ Tregs (212).

Patients treated with a single dose of 300 mg/m^2^ Cy experienced a ~20% Treg decrease sustained for 25 days and a decrease in proliferation marker Ki-67, further substantiating the loss of Treg homeostatic proliferation (177). Further studies of Cy in humans showed that end-stage cancer patients treated with metronomic dosing of 100 mg of Cy per day for 7 days for 4 weeks of on/off, such that the cumulative dose of ~777 mg/m^2^ was split between 2 weeks with a week without treatment in between, had greater than 50% decrease in both relative frequency and absolute counts of Tregs at day 30 of treatment. Importantly, treatment caused an increase in CD8^+^ T cell and NK cell cytotoxicity, a requirement for adequate clearance of infected cells during HIV cure approaches. Interestingly, when the dose was increased to 200 mg/day, the selective depletion of Tregs was ablated, underpinning the importance of the low dose for specific Treg targeting (178).

To be an effective therapy for HIV, Cy must be effective in depleting Tregs from the LNs, where there is a major viral reservoir. A study in mice showed that Cy treatment was beneficial in the LNs by selectively depleting the CD8^+^ lymphoid-resident DCs while sparing the skin-derived migratory DCs and pDCs in the LNs and spleen. This selective depletion in turn boosted antigen presentation and cytokine secretion by the mDCs and pDCs, with a reduction in Treg suppressive capabilities. These results were confirmed by an adoptive transfer of CD8^+^ DCs, which abrogated the immune enhancement (213). When patients were treated with a single IV low-dose of 300 mg/m^2^ Cy, a less immunosuppressive environment compared to controls was observed in the LNs, including significant decreases in IL-10, IL-6, and VEGF (214). Altogether, these results demonstrate that Cy administration is effective in modulating Tregs from both the LNs and periphery.

Little is known about Cy as a therapeutic approach for HIV cure. In an HIV-positive patient with SLE, treatment with Cy induced an enormous burst in viral replication, with plasma viral loads peaking to >1.3 × 10^7^ copies/mL and quickly returned to below detectable levels (215). Using escalating doses up to 1.6 g/m^2^, Bartlett et al., monitored the effects of Cy on HIV DNA in LNs and PBMCs and plasma viral loads. They found no significant difference in the HIV DNA burden of LNs and PBMCs *versus* the control group, but, of note, plasma viral loads were not suppressed in these patients, with two subjects out of five admitting to nonadherence to ART (216). Thus, it is possible that the increase in plasma viral loads and lack of viral DNA clearance may have been due to nonadherence.

Based on these data and the impressive benefits of Cy during various cancer treatments, low-dose Cy could be an effective therapy to decrease HIV reservoir, through its Treg-depleting effect. However, further studies are necessary to detail the potential of Cy to enhance HIV-specific CTL responses and/or reactivate latent HIV.

## Therapies Targeting Treg Function

Various therapies to affect Treg function have also been tested. CTLA-4^+^PD-1^neg^ CD4^+^ T cells from multiple tissues are enriched for replication-competent SIV in infected RMs under ART, suggesting a potential therapeutic target for reservoir elimination (217). During HIV infection, CTLA-4 plays a role in suppression of HIV-specific T cells, with CTLA-4 blockade enhancing CD4^+^ T cell functionality, i.e., IFN-γ production and cell proliferation (218, 219). In an HIV-infected individual treated with Ipilimumab (α-CTLA-4 mAb) for melanoma, plasma viral loads remained below the limit of detection using standard qPCR methods, whereas a general decline in plasma viral loads was seen when using the single copy assay, with an opposing increase in cell-associated unspliced RNA post-treatment, likely due to expansion of infected T cells (179). Additionally, in chronically infected RM given blocking CTLA-4 Ab while on ART, decreases in viral RNA was noted when therapy was interrupted, along with an increase in the SIV-specific immune response (180). However, when the same blockade was used in early infection with the pathogenic SIVmac251-infected RM model, it increased immune activation, viral replication, but did not augment SIV-specific responses, and abrogated responsiveness to ART (154). Thus, further studies need to be conducted to determine whether stand-alone CTLA-4 blockade can be used as a latency reversing strategy.

As mentioned earlier, Tregs also express PD-1, which affected their homeostasis (17, 220). PD-1 also is thought to participate in Treg suppression (221). Due to its major role in contribution to T cell exhaustion (222, 223), efficiency of PD-1/PD-L1 blockade is widely studied in HIV-1 infection. However, the lack of specificity to Tregs of these interventions puts PD-1 targeting out of the scope of this review [reviewed in Ref. (224)].

Indoleamine 2,3-dioxygenase has been observed to increase during HIV infection and may suppress the antiviral responses (225). Thus, IDO blockade has been attempted to enhance the antiviral response to HIV/SIV (181, 182). In SIV-infected macaques under ART, treatment with the IDO inhibitor 1-methyl-d-tryptophan (d-1mT) reduced plasma viral loads and SIV RNA in LNs (181). d-1mT combined with CTLA-4 blockade in SIVmac251-infected macaques under ART did not provide better control of viremia (182). Additionally, this treatment induced acute pancreatitis in all animals, whereas the same ART regimen given alone induced pancreatitis in only 10–20% of the animals (182). These data suggest either an exacerbation of ART toxicity, or more likely, the induction of auto-immune responses against pancreatic antigens. Whatever the underlying mechanisms, such findings are a cautionary warning of the potential risk of any Treg manipulation *in vivo*.

## Conclusion

Treg suppression of virus-specific immune responses may limit the efficacy of virus reactivation strategies, which require effective killing of the reactivated HIV/SIV reservoir. As a result, Tregs may play a central role in shaping the HIV reservoir and compromising the HIV/SIV-specific immune responses. Future research should focus on further refine the effects of various Treg manipulation techniques on the reservoir. Here, we have described several promising Treg treatments that may either suppress Treg activity or kill Tregs altogether. Treg depletion, which has the ability to directly target a small fraction of the reservoir, reactivate the virus, and boost cell-mediated immune responses, might be a desirable strategy for cure research. Although standalone Treg manipulations are promising, they can quite easily be added to other regimens. In the future, investigations into combining Treg therapies with the more traditional viral reactivation therapies, i.e., HDACis, PKA agonists, etc., or vaccinations may prove to be valid cure strategies.

## Author Contributions

Outlined the manuscript (AK, RS, IP, CC, CA); drafted the manuscript (AK, RS); reviewed the manuscript (IP, CC, CA); Overseen the process (CC, CA); prepared the figure (RS, AK). AK, and RS equally contributed to this manuscript. CC and CA are corresponding authors.

## Conflict of Interest Statement

The authors declare that the research was conducted in the absence of any commercial or financial relationships that could be construed as a potential conflict of interest. The reviewer MP and handling Editor declared their shared affiliation.

## References

[1] Gershon RK Kondo K Cell interactions in the induction of tolerance: the role of thymic lymphocytes. Immunology (1970) 18:723–37. 4911896 PMC1455602

[2] Sakaguchi S Sakaguchi N Asano M Itoh M Toda M. Immunologic self-tolerance maintained by activated T cells expressing IL-2 receptor alpha-chains (CD25). Breakdown of a single mechanism of self-tolerance causes various autoimmune diseases. J Immunol (1995) 155:1151–64. 7636184

[3] Lee HM Bautista JL Hsieh CS. Thymic and peripheral differentiation of regulatory T cells. Adv Immunol (2011) 112:25–71. 10.1016/B978-0-12-387827-4.00002-4 22118406

[4] Zhang H Kong H Zeng X Guo L Sun X He S. Subsets of regulatory T cells and their roles in allergy. J Transl Med (2014) 12:125. 10.1186/1479-5876-12-125 24886492 PMC4023533

[5] Mason GM Lowe K Melchiotti R Ellis R de Rinaldis E Peakman M Phenotypic complexity of the human regulatory T cell compartment revealed by mass cytometry. J Immunol (2015) 195:2030–7. 10.4049/jimmunol.1500703 26223658

[6] Yagi H Nomura T Nakamura K Yamazaki S Kitawaki T Hori S Crucial role of FOXP3 in the development and function of human CD25+CD4+ regulatory T cells. Int Immunol (2004) 16:1643–56. 10.1093/intimm/dxh165 15466453

[7] Brunkow ME Jeffery EW Hjerrild KA Paeper B Clark LB Yasayko S-A Disruption of a new forkhead/winged-helix protein, scurfin, results in the fatal lymphoproliferative disorder of the scurfy mouse. Nat Genet (2001) 27:68. 10.1038/83784 11138001

[8] Huter EN Punkosdy GA Glass DD Cheng LI Ward JM Shevach EM TGFβ-induced FoxP3(+) regulatory T cells rescue scurfy mice. Eur J Immunol (2008) 38:1814–21. 10.1002/eji.200838346 18546144 PMC2574868

[9] Liu W Putnam AL Xu-Yu Z Szot GL Lee MR Zhu S CD127 expression inversely correlates with FoxP3 and suppressive function of human CD4+ T reg cells. J Exp Med (2006) 203:1701–11. 10.1084/jem.20060772 16818678 PMC2118339

[10] Yu N Li X Song W Li D Yu D Zeng X CD4+CD25+CD127low/- T cells: a more specific Treg population in human peripheral blood. Inflammation (2012) 35:1773–80. 10.1007/s10753-012-9496-8 22752562

[11] Mandelbrot DA McAdam AJ Sharpe AH B7-1 or B7-2 is required to produce the lymphoproliferative phenotype in mice lacking cytotoxic T lymphocyte-associated antigen 4 (CTLA-4). J Exp Med (1999) 189:435–40. 10.1084/jem.189.2.435 9892625 PMC2192978

[12] Krummel MF Allison JP. CTLA-4 engagement inhibits IL-2 accumulation and cell cycle progression upon activation of resting T cells. J Exp Med (1996) 183:2533–40. 10.1084/jem.183.6.2533 8676074 PMC2192613

[13] Tivol EA Borriello F Schweitzer AN Lynch WP Bluestone JA Sharpe AH. Loss of CTLA-4 leads to massive lymphoproliferation and fatal multiorgan tissue destruction, revealing a critical negative regulatory role of CTLA-4. Immunity (1995) 3:541–7. 10.1016/1074-7613(95)90125-6 7584144

[14] Wing K Onishi Y Prieto-Martin P Yamaguchi T Miyara M Fehervari Z CTLA-4 control over Foxp3+ regulatory T cell function. Science (2008) 322:271–5. 10.1126/science.1160062 18845758

[15] Guo F Iclozan C Suh W-K Anasetti C Yu X-Z. CD28 controls differentiation of regulatory T cells from naive CD4 T cells. J Immunol (2008) 181:2285–91. 10.4049/jimmunol.181.4.2285 18684917 PMC2688779

[16] Zhang R Huynh A Whitcher G Chang J Maltzman JS Turka LA. An obligate cell-intrinsic function for CD28 in Tregs. J Clin Invest (2013) 123:580–93. 10.1172/JCI65013 23281398 PMC3561819

[17] Asano T Kishi Y Meguri Y Yoshioka T Iwamoto M Maeda Y PD-1 signaling has a critical role in maintaining regulatory T cell homeostasis; implication for Treg depletion therapy by PD-1 blockade. Blood (2015) 126:848–848.

[18] Freeman GJ Long AJ Iwai Y Bourque K Chernova T Nishimura H Engagement of the PD-1 immunoinhibitory receptor by a novel B7 family member leads to negative regulation of lymphocyte activation. J Exp Med (2000) 192:1027–34. 10.1084/jem.192.7.1027 11015443 PMC2193311

[19] Zhang B Chikuma S Hori S Fagarasan S Honjo T. Nonoverlapping roles of PD-1 and FoxP3 in maintaining immune tolerance in a novel autoimmune pancreatitis mouse model. Proc Natl Acad Sci U S A (2016) 113:8490–5. 10.1073/pnas.1608873113 27410049 PMC4968716

[20] Vocanson M Rozieres A Hennino A Poyet G Gaillard V Renaudineau S Inducible costimulator (ICOS) is a marker for highly suppressive antigen-specific T cells sharing features of TH17/TH1 and regulatory T cells. J Allergy Clin Immunol (2010) 126:280–9, 289.e1–7. 10.1016/j.jaci.2010.05.022 20624644

[21] Redpath SA van der Werf N Cervera AM MacDonald AS Gray D Maizels RM ICOS controls Foxp3(+) regulatory T-cell expansion, maintenance and IL-10 production during helminth infection. Eur J Immunol (2013) 43:705–15. 10.1002/eji.201242794 23319295 PMC3615169

[22] Ito T Hanabuchi S Wang YH Park WR Arima K Bover L Two functional subsets of FOXP3+ regulatory T cells in human thymus and periphery. Immunity (2008) 28:870–80. 10.1016/j.immuni.2008.03.018 18513999 PMC2709453

[23] Do J-S Visperas A Sanogo YO Bechtel JJ Dvorina N Kim S An IL-27/Lag3 axis enhances Foxp3(+) regulatory T cell suppressive function and therapeutic efficacy. Mucosal Immunol (2016) 9:137–45. 10.1038/mi.2015.45 26013006 PMC4662649

[24] Ronchetti S Ricci E Petrillo MG Cari L Migliorati G Nocentini G Glucocorticoid-induced tumour necrosis factor receptor-related protein: a key marker of functional regulatory T cells. J Immunol Res (2015) 2015:171520. 10.1155/2015/171520 25961057 PMC4413981

[25] Liao G Nayak S Regueiro JR Berger SB Detre C Romero X GITR engagement preferentially enhances proliferation of functionally competent CD4+CD25+FoxP3+ regulatory T cells. Int Immunol (2010) 22:259–70. 10.1093/intimm/dxq001 20139172 PMC2845330

[26] Sun L Jin H Li H GARP: a surface molecule of regulatory T cells that is involved in the regulatory function and TGF-beta releasing. Oncotarget (2016) 7:42826–36. 10.18632/oncotarget.8753 27095576 PMC5173174

[27] Tran DQ Andersson J Wang R Ramsey H Unutmaz D Shevach EM. GARP (LRRC32) is essential for the surface expression of latent TGF-beta on platelets and activated FOXP3+ regulatory T cells. Proc Natl Acad Sci U S A (2009) 106:13445–50. 10.1073/pnas.0901944106 19651619 PMC2726354

[28] Chen X Wu X Zhou Q Howard OM Netea MG Oppenheim JJ. TNFR2 is critical for the stabilization of the CD4+Foxp3+ regulatory T. cell phenotype in the inflammatory environment. J Immunol (2013) 190:1076–84. 10.4049/jimmunol.1202659 23277487 PMC3552130

[29] Takatori H Kawashima H Matsuki A Meguro K Tanaka S Iwamoto T Helios enhances Treg cell function in cooperation with FoxP3. Arthritis Rheumatol (2015) 67:1491–502. 10.1002/art.39091 25733061

[30] Sebastian M Lopez-Ocasio M Metidji A Rieder SA Shevach EM Thornton AM. Helios controls a limited subset of regulatory T cell functions. J Immunol (2016) 196:144–55. 10.4049/jimmunol.1501704 26582951 PMC4685018

[31] Thornton AM Kilaru G Burr P Rieder S Muljo SA Shevach EM Helios expression defines two distinct populations of Foxp3+ regulatory T cells. J Immunol (2016) 196:125–6.

[32] Singh K Hjort M Thorvaldson L Sandler S. Concomitant analysis of helios and neuropilin-1 as a marker to detect thymic derived regulatory T cells in naïve mice. Sci Rep (2015) 5:7767. 10.1038/srep07767 25586548 PMC4293597

[33] Borsellino G Kleinewietfeld M Di Mitri D Sternjak A Diamantini A Giometto R Expression of ectonucleotidase CD39 by Foxp3+ Treg cells: hydrolysis of extracellular ATP and immune suppression. Blood (2007) 110:1225–32. 10.1182/blood-2006-12-064527 17449799

[34] He T Brocca-Cofano E Gillespie DG Xu C Stock JL Ma D Critical role for the adenosine pathway in controlling simian immunodeficiency virus-related immune activation and inflammation in gut mucosal tissues. J Virol (2015) 89:9616–30. 10.1128/JVI.01196-15 26178986 PMC4542384

[35] Deaglio S Dwyer KM Gao W Friedman D Usheva A Erat A Adenosine generation catalyzed by CD39 and CD73 expressed on regulatory T cells mediates immune suppression. J Exp Med (2007) 204:1257–65. 10.1084/jem.20062512 17502665 PMC2118603

[36] Sugiyama D Nishikawa H Maeda Y Nishioka M Tanemura A Katayama I Anti-CCR4 mAb selectively depletes effector-type FoxP3+CD4+ regulatory T cells, evoking antitumor immune responses in humans. Proc Natl Acad Sci U S A (2013) 110:17945–50. 10.1073/pnas.1316796110 24127572 PMC3816454

[37] Curiel TJ Coukos G Zou L Alvarez X Cheng P Mottram P Specific recruitment of regulatory T cells in ovarian carcinoma fosters immune privilege and predicts reduced survival. Nat Med (2004) 10:942–9. 10.1038/nm1093 15322536

[38] Lee I Wang L Wells AD Dorf ME Ozkaynak E Hancock WW. Recruitment of Foxp3+ T regulatory cells mediating allograft tolerance depends on the CCR4 chemokine receptor. J Exp Med (2005) 201:1037–44. 10.1084/jem.20041709 15809349 PMC2213137

[39] Yamazaki T Yang XO Chung Y Fukunaga A Nurieva R Pappu B CCR6 regulates the migration of inflammatory and regulatory T cells. J Immunol (2008) 181:8391–401. 10.4049/jimmunol.181.12.8391 19050256 PMC2752441

[40] Schneider MA Meingassner JG Lipp M Moore HD Rot A. CCR7 is required for the in vivo function of CD4+ CD25+ regulatory T cells. J Exp Med (2007) 204:735–45. 10.1084/jem.20061405 17371928 PMC2118557

[41] Tosello V Odunsi K Souleimanian NE Lele S Shrikant P Old LJ Differential expression of CCR7 defines two distinct subsets of human memory CD4+CD25+ Tregs. Clin Immunol (2008) 126:291–302. 10.1016/j.clim.2007.11.008 18166500

[42] Cowan JE McCarthy NI Anderson G. CCR7 controls thymus recirculation, but not production and emigration, of Foxp3(+) T cells. Cell Rep (2016) 14:1041–8. 10.1016/j.celrep.2016.01.003 26832402 PMC4751304

[43] Chung Y Tanaka S Chu F Nurieva RI Martinez GJ Rawal S Follicular regulatory T cells expressing Foxp3 and Bcl-6 suppress germinal center reactions. Nat Med (2011) 17:983–8. 10.1038/nm.2426 21785430 PMC3151340

[44] Collison LW Pillai MR Chaturvedi V Vignali DAA. Regulatory T cell suppression is potentiated by target T cells in a cell contact, IL-35- and IL-10-dependent manner. J Immunol (2009) 182:6121–8. 10.4049/jimmunol.0803646 19414764 PMC2698997

[45] Strauss L Bergmann C Szczepanski M Gooding W Johnson JT Whiteside TL. A unique subset of CD4+CD25highFoxp3+ T cells secreting interleukin-10 and transforming growth factor-beta1 mediates suppression in the tumor microenvironment. Clin Cancer Res (2007) 13:4345–54. 10.1158/1078-0432.CCR-07-0472 17671115

[46] Li MO Wan YY Flavell RA T cell-produced transforming growth factor-β1 controls T cell tolerance and regulates Th1- and Th17-cell differentiation. Immunity (2007) 26:579–91. 10.1016/j.immuni.2007.03.014 17481928

[47] Gil-Guerrero L Dotor J Huibregtse IL Casares N López-Vázquez AB Rudilla F In vitro and in vivo down-regulation of regulatory T cell activity with a peptide inhibitor of TGF-β1. J Immunol (2008) 181:126–35. 10.4049/jimmunol.181.1.126 18566377

[48] Shen E Zhao K Wu C Yang B The suppressive effect of CD25+Treg cells on Th1 differentiation requires cell-cell contact partially via TGF-beta production. Cell Biol Int (2011) 35:705–12. 10.1042/CBI20100528 21314640

[49] Ahmadzadeh M Rosenberg SA. TGF-beta 1 attenuates the acquisition and expression of effector function by tumor antigen-specific human memory CD8 T cells. J Immunol (2005) 174:5215–23. 10.4049/jimmunol.174.9.5215 15843517 PMC2562293

[50] Konkel JE Zhang D Zanvit P Chia C Zangarle-Murray T Jin W Transforming growth factor-beta signaling in regulatory T cells controls T helper-17 cells and tissue-specific immune responses. Immunity (2017) 46:660–74. 10.1016/j.immuni.2017.03.015 28423340 PMC12230991

[51] Collison LW Workman CJ Kuo TT Boyd K Wang Y Vignali KM The inhibitory cytokine IL-35 contributes to regulatory T-cell function. Nature (2007) 450:566. 10.1038/nature06306 18033300

[52] Wang J Ioan-Facsinay A van der Voort EI Huizinga TW Toes RE. Transient expression of FOXP3 in human activated nonregulatory CD4+ T cells. Eur J Immunol (2007) 37:129–38. 10.1002/eji.200636435 17154262

[53] Jordan MS Boesteanu A Reed AJ Petrone AL Holenbeck AE Lerman MA Thymic selection of CD4+CD25+ regulatory T cells induced by an agonist self-peptide. Nat Immunol (2001) 2:301. 10.1038/86302 11276200

[54] Weissler KA Caton AJ The role of T-cell receptor recognition of peptide:MHC complexes in the formation and activity of Foxp3+ regulatory T cells. Immunol Rev (2014) 259:11–22. 10.1111/imr.12177 24712456 PMC4034456

[55] Toker A Engelbert D Garg G Polansky JK Floess S Miyao T Active demethylation of the Foxp3 locus leads to the generation of stable regulatory T cells within the thymus. J Immunol (2013) 190:3180–8. 10.4049/jimmunol.1203473 23420886

[56] Ohkura N Hamaguchi M Morikawa H Sugimura K Tanaka A Ito Y T cell receptor stimulation-induced epigenetic changes and Foxp3 expression are independent and complementary events required for Treg cell development. Immunity (2012) 37:785–99. 10.1016/j.immuni.2012.09.010 23123060

[57] Zhou P Zheng X Zhang H Liu Y Zheng P. B7 blockade alters the balance between regulatory T cells and tumor-reactive T cells for immunotherapy of cancer. Clin Cancer Res (2009) 15:960–70. 10.1158/1078-0432.CCR-08-1611 19188167 PMC2693886

[58] Tang Q Henriksen KJ Boden EK Tooley AJ Ye J Subudhi SK Cutting edge: CD28 controls peripheral homeostasis of CD4+CD25+ regulatory T cells. J Immunol (2003) 171:3348–52. 10.4049/jimmunol.171.7.3348 14500627

[59] Cheng G Yu A Dee MJ Malek TR. IL-2R signaling is essential for functional maturation of regulatory T cells during thymic development. J Immunol (2013) 190:1567–75. 10.4049/jimmunol.1201218 23315074 PMC3563871

[60] Fu S Zhang N Yopp AC Chen D Mao M Chen D TGF-β induces Foxp3 + T-regulatory cells from CD4 + CD25 − precursors. Am J Transplant (2004) 4:1614–27. 10.1111/j.1600-6143.2004.00566.x 15367216

[61] Chen W Jin W Hardegen N Lei K-J Li L Marinos N Conversion of peripheral CD4+CD25− naive T cells to CD4+CD25+ regulatory T cells by TGF-β induction of transcription factor Foxp3. J Exp Med (2003) 198:1875–86. 10.1084/jem.20030152 14676299 PMC2194145

[62] Polansky JK Kretschmer K Freyer J Floess S Garbe A Baron U DNA methylation controls Foxp3 gene expression. Eur J Immunol (2008) 38:1654–63. 10.1002/eji.200838105 18493985

[63] Coombes JL Siddiqui KR Arancibia-Cárcamo CV Hall J Sun CM Belkaid Y A functionally specialized population of mucosal CD103+ DCs induces Foxp3+ regulatory T cells via a TGF-beta and retinoic acid-dependent mechanism. J Exp Med (2007) 204:1757–64. 10.1084/jem.20070590 17620361 PMC2118683

[64] Mucida D Park Y Kim G Turovskaya O Scott I Kronenberg M Reciprocal TH17 and regulatory T cell differentiation mediated by retinoic acid. Science (2007) 317:256–60. 10.1126/science.1145697 17569825

[65] Xiao S Jin H Korn T Liu SM Oukka M Lim B Retinoic acid increases Foxp3+ regulatory T cells and inhibits development of Th17 cells by enhancing TGF-β-driven Smad3 signaling and inhibiting IL-6 and IL-23 receptor expression. J Immunol (2008) 181:2277–84. 10.4049/jimmunol.181.4.2277 18684916 PMC2722959

[66] Bastien J Plassat JL Payrastre B Rochette-Egly C The phosphoinositide 3-kinase/Akt pathway is essential for the retinoic acid-induced differentiation of F9 cells. Oncogene (2005) 25:2040 10.1038/sj.onc.1209241 16288212

[67] Haxhinasto S Mathis D Benoist C The AKT–mTOR axis regulates de novo differentiation of CD4+Foxp3+ cells. J Exp Med (2008) 205:565–74. 10.1084/jem.20071477 18283119 PMC2275380

[68] Apostolou I von Boehmer H. In vivo instruction of suppressor commitment in naive T cells. J Exp Med (2004) 199:1401–8. 10.1084/jem.20040249 15148338 PMC2211808

[69] D’Cruz LM Klein L. Development and function of agonist-induced CD25+Foxp3+ regulatory T cells in the absence of interleukin 2 signaling. Nat Immunol (2005) 6:1152–9. 10.1038/ni1264 16227983

[70] Fontenot JD Rasmussen JP Gavin MA Rudensky AY. A function for interleukin 2 in Foxp3-expressing regulatory T cells. Nat Immunol (2005) 6:1142–51. 10.1038/ni1263 16227984

[71] Smigiel KS Richards E Srivastava S Thomas KR Dudda JC Klonowski KD CCR7 provides localized access to IL-2 and defines homeostatically distinct regulatory T cell subsets. J Exp Med (2014) 211:121–36. 10.1084/jem.20131142 24378538 PMC3892972

[72] Walker LS Chodos A Eggena M Dooms H Abbas AK. Antigen-dependent proliferation of CD4+ CD25+ regulatory T cells in vivo. J Exp Med (2003) 198:249–58. 10.1084/jem.20030315 12874258 PMC2194065

[73] Salomon B Lenschow DJ Rhee L Ashourian N Singh B Sharpe A B7/CD28 costimulation is essential for the homeostasis of the CD4+CD25+ immunoregulatory T cells that control autoimmune diabetes. Immunity (2000) 12:431–40. 10.1016/S1074-7613(00)80195-8 10795741

[74] Fu S Yopp AC Mao X Chen D Zhang N Chen D CD4+ CD25+ CD62+ T-regulatory cell subset has optimal suppressive and proliferative potential. Am J Transplant (2004) 4:65–78. 10.1046/j.1600-6143.2003.00293.x 14678036

[75] Ochando JC Yopp AC Yang Y Garin A Li Y Boros P Lymph node occupancy is required for the peripheral development of alloantigen-specific Foxp3+ regulatory T cells. J Immunol (2005) 174:6993–7005. 10.4049/jimmunol.174.11.6993 15905542

[76] Wei S Kryczek I Zou W. Regulatory T-cell compartmentalization and trafficking. Blood (2006) 108:426–31. 10.1182/blood-2006-01-0177 16537800 PMC1895488

[77] Lim HW Hillsamer P Kim CH Regulatory T cells can migrate to follicles upon T cell activation and suppress GC-Th cells and GC-Th cell-driven B cell responses. J Clin Invest (2004) 114:1640–9. 10.1172/JCI200422325 15578096 PMC529283

[78] Linterman MA Pierson W Lee SK Kallies A Kawamoto S Rayner TF Foxp3+ follicular regulatory T cells control the germinal center response. Nat Med (2011) 17:975–82. 10.1038/nm.2425 21785433 PMC3182542

[79] Wollenberg I Agua-Doce A Hernandez A Almeida C Oliveira VG Faro J Regulation of the germinal center reaction by Foxp3+ follicular regulatory T cells. J Immunol (2011) 187:4553–60. 10.4049/jimmunol.1101328 21984700

[80] Alexander C-M Tygrett LT Boyden AW Wolniak KL Legge KL Waldschmidt TJ. T regulatory cells participate in the control of germinal centre reactions. Immunology (2011) 133:452–68. 10.1111/j.1365-2567.2011.03456.x 21635248 PMC3143357

[81] Wing James B Ise W Kurosaki T Sakaguchi S. Regulatory T cells control antigen-specific expansion of Tfh cell number and humoral immune responses via the coreceptor CTLA-4. Immunity (2014) 41:1013–25. 10.1016/j.immuni.2014.12.006 25526312

[82] Sage PT Alvarez D Godec J von Andrian UH Sharpe AH. Circulating T follicular regulatory and helper cells have memory-like properties. J Clin Invest (2014) 124:5191–204. 10.1172/JCI76861 25347469 PMC4348955

[83] Sage PT Sharpe AH. T follicular regulatory cells in the regulation of B cell responses. Trends Immunol (2015) 36:410–8. 10.1016/j.it.2015.05.005 26091728 PMC4508020

[84] Wing JB Kitagawa Y Locci M Hume H Tay C Morita T A distinct subpopulation of CD25− T-follicular regulatory cells localizes in the germinal centers. Proc Natl Acad Sci U S A (2017) 114:E6400–9. 10.1073/pnas.1705551114 28698369 PMC5547636

[85] Morgan ME Sutmuller RP Witteveen HJ van Duivenvoorde LM Zanelli E Melief CJ CD25+ cell depletion hastens the onset of severe disease in collagen-induced arthritis. Arthritis Rheum (2003) 48:1452–60. 10.1002/art.11063 12746920

[86] Eddahri F Oldenhove G Denanglaire S Urbain J Leo O Andris F. CD4+ CD25+ regulatory T cells control the magnitude of T-dependent humoral immune responses to exogenous antigens. Eur J Immunol (2006) 36:855–63. 10.1002/eji.200535500 16511897

[87] Tang Q Adams JY Tooley AJ Bi M Fife BT Serra P Visualizing regulatory T cell control of autoimmune responses in nonobese diabetic mice. Nat Immunol (2005) 7:83 10.1038/ni1289 16311599 PMC3057888

[88] Milanez-Almeida P Meyer-Hermann M Toker A Khailaie S Huehn J. Foxp3+ regulatory T-cell homeostasis quantitatively differs in murine peripheral lymph nodes and spleen. Eur J Immunol (2015) 45:153–66. 10.1002/eji.201444480 25330759

[89] Takahashi T Kuniyasu Y Toda M Sakaguchi N Itoh M Iwata M Immunologic self-tolerance maintained by CD25+CD4+ naturally anergic and suppressive T cells: induction of autoimmune disease by breaking their anergic/suppressive state. Int Immunol (1998) 10:1969–80. 10.1093/intimm/10.12.1969 9885918

[90] Massague J Andres J Attisano L Cheifetz S Lopez-Casillas F Ohtsuki M TGF-beta receptors. Mol Reprod Dev (1992) 32:99–104. 1322148 10.1002/mrd.1080320204

[91] Nakamura K Kitani A Strober W Cell contact-dependent immunosuppression by Cd4+Cd25+regulatory T cells is mediated by cell surface-bound transforming growth factor β. J Exp Med (2001) 194:629–44. 10.1084/jem.194.5.629 11535631 PMC2195935

[92] Rubtsov YP Rasmussen JP Chi EY Fontenot J Castelli L Ye X Regulatory T cell-derived interleukin-10 limits inflammation at environmental interfaces. Immunity (2008) 28:546–58. 10.1016/j.immuni.2008.02.017 18387831

[93] Collison LW Chaturvedi V Henderson AL Giacomin PR Guy C Bankoti J IL-35-mediated induction of a potent regulatory T cell population. Nat Immunol (2010) 11:1093. 10.1038/ni.1952 20953201 PMC3008395

[94] Bardel E Larousserie F Charlot-Rabiega P Coulomb-L’Hermine A Devergne O. Human CD4+CD25+Foxp3+ regulatory T cells do not constitutively express IL-35. J Immunol (2008) 181:6898–905. 10.4049/jimmunol.181.10.6898 18981109

[95] Mellor AL Chandler P Baban B Hansen AM Marshall B Pihkala J Specific subsets of murine dendritic cells acquire potent T cell regulatory functions following CTLA4-mediated induction of indoleamine 2,3 dioxygenase. Int Immunol (2004) 16:1391–401. 10.1093/intimm/dxh140 15351783

[96] Takahashi T Tagami T Yamazaki S Uede T Shimizu J Sakaguchi N Immunologic self-tolerance maintained by Cd25+Cd4+regulatory T cells constitutively expressing cytotoxic T lymphocyte-associated antigen 4. J Exp Med (2000) 192:303–10. 10.1084/jem.192.2.303 10899917 PMC2193248

[97] Tivol EA Boyd SD McKeon S Borriello F Nickerson P Strom TB CTLA4Ig prevents lymphoproliferation and fatal multiorgan tissue destruction in CTLA-4-deficient mice. J Immunol (1997) 158:5091–4. 9164923

[98] Matheu MP Othy S Greenberg ML Dong TX Schuijs M Deswarte K Imaging regulatory T cell dynamics and CTLA4-mediated suppression of T cell priming. Nat Commun (2015) 6:6219. 10.1038/ncomms7219 25653051 PMC4347855

[99] Carreno BM Bennett F Chau TA Ling V Luxenberg D Jussif J CTLA-4 (CD152) can inhibit T cell activation by two different mechanisms depending on its level of cell surface expression. J Immunol (2000) 165:1352–6. 10.4049/jimmunol.165.3.1352 10903737

[100] Romano E Kusio-Kobialka M Foukas PG Baumgaertner P Meyer C Ballabeni P Ipilimumab-dependent cell-mediated cytotoxicity of regulatory T cells ex vivo by nonclassical monocytes in melanoma patients. Proc Natl Acad Sci U S A (2015) 112:6140–5. 10.1073/pnas.1417320112 25918390 PMC4434760

[101] Thornton AM Shevach EM. CD4+CD25+immunoregulatory T cells suppress polyclonal T cell activation in vitro by inhibiting interleukin 2 production. J Exp Med (1998) 188:287–96. 10.1084/jem.188.2.287 9670041 PMC2212461

[102] Pandiyan P Zheng L Ishihara S Reed J Lenardo MJ CD4+CD25+Foxp3+ regulatory T cells induce cytokine deprivation-mediated apoptosis of effector CD4+ T cells. Nat Immunol (2007) 8:1353 10.1038/ni1536 17982458

[103] de la Rosa M Rutz S Dorninger H Scheffold A. Interleukin-2 is essential for CD4+CD25+ regulatory T cell function. Eur J Immunol (2004) 34:2480–8. 10.1002/eji.200425274 15307180

[104] Barthlott T Moncrieffe H Veldhoen M Atkins CJ Christensen J O’Garra A CD25+ CD4+ T cells compete with naive CD4+ T cells for IL-2 and exploit it for the induction of IL-10 production. Int Immunol (2005) 17:279–88. 10.1093/intimm/dxh207 15684039

[105] Cekic C Linden J Purinergic regulation of the immune system. Nat Rev Immunol (2016) 16:177 10.1038/nri.2016.4 26922909

[106] Rissiek A Baumann I Cuapio A Mautner A Kolster M Arck PC The expression of CD39 on regulatory T cells is genetically driven and further upregulated at sites of inflammation. J Autoimmun (2015) 58:12–20. 10.1016/j.jaut.2014.12.007 25640206

[107] Nikolova M Carriere M Jenabian M-A Limou S Younas M Kök A CD39/adenosine pathway is involved in AIDS progression. PLoS Pathog (2011) 7:e1002110. 10.1371/journal.ppat.1002110 21750674 PMC3131268

[108] Langer D Hammer K Koszalka P Schrader J Robson S Zimmermann H. Distribution of ectonucleotidases in the rodent brain revisited. Cell Tissue Res (2008) 334:199–217. 10.1007/s00441-008-0681-x 18843508

[109] Kobie JJ Shah PR Yang L Rebhahn JA Fowell DJ Mosmann TR T regulatory and primed uncommitted CD4 T cells express CD73, which suppresses effector CD4 T cells by converting 5′-adenosine monophosphate to adenosine. J Immunol (2006) 177:6780–6. 10.4049/jimmunol.177.10.6780 17082591

[110] Mandapathil M Hilldorfer B Szczepanski MJ Czystowska M Szajnik M Ren J Generation and accumulation of immunosuppressive adenosine by human CD4+CD25highFOXP3+ regulatory T cells. J Biol Chem (2010) 285:7176–86. 10.1074/jbc.M109.047423 19858205 PMC2844167

[111] Huang S Apasov S Koshiba M Sitkovsky M. Role of A2a extracellular adenosine receptor-mediated signaling in adenosine-mediated inhibition of T-cell activation and expansion. Blood (1997) 90:1600–10. 9269779

[112] Lappas CM Rieger JM Linden J A2A adenosine receptor induction inhibits IFN-γ production in murine CD4+ T cells. J Immunol (2005) 174:1073–80. 10.4049/jimmunol.174.2.1073 15634932

[113] Panther E Corinti S Idzko M Herouy Y Napp M la Sala A Adenosine affects expression of membrane molecules, cytokine and chemokine release, and the T-cell stimulatory capacity of human dendritic cells. Blood (2003) 101:3985–90. 10.1182/blood-2002-07-2113 12446452

[114] Klein M Bopp T. Cyclic AMP represents a crucial component of Treg cell-mediated immune regulation. Front Immunol (2016) 7:315. 10.3389/fimmu.2016.00315 27621729 PMC5002888

[115] Bopp T Becker C Klein M Klein-Hessling S Palmetshofer A Serfling E Cyclic adenosine monophosphate is a key component of regulatory T cell-mediated suppression. J Exp Med (2007) 204:1303–10. 10.1084/jem.20062129 17502663 PMC2118605

[116] Moreno-Fernandez ME Rueda CM Rusie LK Chougnet CA. Regulatory T cells control HIV replication in activated T cells through a cAMP-dependent mechanism. Blood (2011) 117:5372–80. 10.1182/blood-2010-12-323162 21436067 PMC3109711

[117] Moreno-Fernandez ME Joedicke JJ Chougnet CA Regulatory T cells diminish HIV infection in dendritic cells – conventional CD4(+) T cell clusters. Front Immunol (2014) 5:199 10.3389/fimmu.2014.00199 24847325 PMC4021135

[118] Estes JD Li Q Reynolds MR Wietgrefe S Duan L Schacker T Premature induction of an immunosuppressive regulatory T cell response during acute simian immunodeficiency virus infection. J Infect Dis (2006) 193:703–12. 10.1086/500368 16453267

[119] Schulze zur Wiesch J Thomssen A Hartjen P Tóth I Lehmann C Meyer-Olson D Comprehensive analysis of frequency and phenotype of T regulatory cells in HIV infection: CD39 expression of FoxP3+ T regulatory cells correlates with progressive disease. J Virol (2011) 85:1287–97. 10.1128/JVI.01758-10 21047964 PMC3020516

[120] Presicce P Orsborn K King E Pratt J Fichtenbaum CJ Chougnet CA. Frequency of circulating regulatory T cells increases during chronic HIV infection and is largely controlled by highly active antiretroviral therapy. PLoS One (2011) 6:e28118. 10.1371/journal.pone.0028118 22162758 PMC3230597

[121] Andersson J Boasso A Nilsson J Zhang R Shire NJ Lindback S Cutting edge: the prevalence of regulatory T cells in lymphoid tissue is correlated with viral load in HIV-infected patients. J Immunol (2005) 174:3143–7. 10.4049/jimmunol.174.6.3143 15749840

[122] Nilsson J Boasso A Velilla PA Zhang R Vaccari M Franchini G HIV-1–driven regulatory T-cell accumulation in lymphoid tissues is associated with disease progression in HIV/AIDS. Blood (2006) 108:3808–17. 10.1182/blood-2006-05-021576 16902147 PMC1895475

[123] Suchard MS Mayne E Green VA Shalekoff S Donninger SL Stevens WS FOXP3 expression is upregulated in CD4(+)T cells in progressive HIV-1 infection and is a marker of disease severity. PLoS One (2010) 5:e11762 10.1371/journal.pone.0011762 20668701 PMC2909259

[124] Tsunemi S Iwasaki T Imado T Higasa S Kakishita E Shirasaka T Relationship of CD4+CD25+ regulatory T cells to immune status in HIV-infected patients. AIDS (2005) 19:879–86. 10.1097/01.aids.0000171401.23243.56 15905668

[125] Eggena MP Barugahare B Jones N Okello M Mutalya S Kityo C Depletion of regulatory T cells in HIV infection is associated with immune activation. J Immunol (2005) 174:4407–14. 10.4049/jimmunol.174.7.4407 15778406

[126] Epple HJ Loddenkemper C Kunkel D Troger H Maul J Moos V Mucosal but not peripheral FOXP3+ regulatory T cells are highly increased in untreated HIV infection and normalize after suppressive HAART. Blood (2006) 108:3072–8. 10.1182/blood-2006-04-016923 16728694

[127] Owen RE Heitman JW Hirschkorn DF Lanteri MC Biswas HH Martin JN HIV(+) elite controllers have low HIV-specific T cell activation yet maintain strong, polyfunctional T cell responses. AIDS (2010) 24:1095–105. 10.1097/QAD.0b013e3283377a1e 20400885 PMC2972651

[128] Allers K Loddenkemper C Hofmann J Unbehaun A Kunkel D Moos V Gut mucosal FOXP3+ regulatory CD4+ T cells and nonregulatory CD4+ T cells are differentially affected by simian immunodeficiency virus infection in Rhesus macaques. J Virol (2010) 84:3259–69. 10.1128/JVI.01715-09 20071575 PMC2838127

[129] Chase AJ Yang H-C Zhang H Blankson JN Siliciano RF. Preservation of FoxP3+ regulatory T cells in the peripheral blood of human immunodeficiency virus type 1-infected elite suppressors correlates with low CD4+ T-cell activation. J Virol (2008) 82:8307–15. 10.1128/JVI.00520-08 18579608 PMC2519624

[130] Becker C Taube C Bopp T Becker C Michel K Kubach J Protection from graft-versus-host disease by HIV-1 envelope protein gp120-mediated activation of human CD4+CD25+ regulatory T cells. Blood (2009) 114:1263–9. 10.1182/blood-2009-02-206730 19439734

[131] Manches O Munn D Fallahi A Lifson J Chaperot L Plumas J HIV-activated human plasmacytoid DCs induce Tregs through an indoleamine 2,3-dioxygenase–dependent mechanism. J Clin Invest (2008) 118:3431–9. 10.1172/JCI34823 18776940 PMC2528911

[132] Presicce P Shaw JM Miller CJ Shacklett BL Chougnet CA Myeloid dendritic cells isolated from tissues of SIV-infected Rhesus macaques promote the induction of regulatory T-cells. AIDS (2012) 26:263–73. 10.1097/QAD.0b013e32834ed8df 22095196 PMC3666583

[133] Estes JD Wietgrefe S Schacker T Southern P Beilman G Reilly C Simian immunodeficiency virus-induced lymphatic tissue fibrosis is mediated by transforming growth factor β1-positive regulatory T cells and begins in early infection. J Infect Dis (2007) 195:551–61. 10.1086/510852 17230415

[134] Shaw JM Hunt PW Critchfield JW McConnell DH Garcia JC Pollard RB Increased frequency of regulatory T cells accompanies increased immune activation in rectal mucosae of HIV-positive noncontrollers. J Virol (2011) 85:11422–34. 10.1128/JVI.05608-11 21880771 PMC3194952

[135] Kinter A McNally J Riggin L Jackson R Roby G Fauci AS Suppression of HIV-specific T cell activity by lymph node CD25(+) regulatory T cells from HIV-infected individuals. Proc Natl Acad Sci U S A (2007) 104:3390–5. 10.1073/pnas.0611423104 17360656 PMC1805624

[136] Chomont N El-Far M Ancuta P Trautmann L Procopio FA Yassine-Diab B HIV reservoir size and persistence are driven by T cell survival and homeostatic proliferation. Nat Med (2009) 15:893–900. 10.1038/nm.1972 19543283 PMC2859814

[137] Estes JD Kityo C Ssali F Swainson L Makamdop KN Del Prete GQ Defining total-body AIDS-virus burden with implications for curative strategies. Nat Med (2017) 23(11):1271–6. 10.1038/nm.4411 28967921 PMC5831193

[138] Cubas RA Mudd JC Savoye A-L Perreau M van Grevenynghe J Metcalf T Inadequate T follicular cell help impairs B cell immunity during HIV infection. Nat Med (2013) 19:494–9. 10.1038/nm.3109 23475201 PMC3843317

[139] Miles B Miller SM Folkvord JM Kimball A Chamanian M Meditz AL Follicular regulatory T cells impair follicular T helper cells in HIV and SIV infection. Nat Commun (2015) 6:8608. 10.1038/ncomms9608 26482032 PMC4616158

[140] Hong JJ Amancha PK Rogers K Ansari AA Villinger F. Spatial alterations between CD4(+) T follicular helper, B, and CD8(+) T cells during simian immunodeficiency virus infection: T/B cell homeostasis, activation, and potential mechanism for viral escape. J Immunol (2012) 188:3247–56. 10.4049/jimmunol.1103138 22387550 PMC3311732

[141] Lindqvist M van Lunzen J Soghoian DZ Kuhl BD Ranasinghe S Kranias G Expansion of HIV-specific T follicular helper cells in chronic HIV infection. J Clin Invest (2012) 122:3271–80. 10.1172/JCI64314 22922259 PMC3428098

[142] Petrovas C Yamamoto T Gerner MY Boswell KL Wloka K Smith EC CD4 T follicular helper cell dynamics during SIV infection. J Clin Invest (2012) 122:3281–94. 10.1172/JCI63039 22922258 PMC3428091

[143] Brocca-Cofano E Kuhrt D Siewe B Xu C Haret-Richter GS Craigo J Pathogenic correlates of the simian immunodeficiency virus (SIV)-associated B cell dysfunction. J Virol (2017) 91 10.1128/jvi.01051-17 PMC5686751 28931679

[144] De Milito A Nilsson A Titanji K Thorstensson R Reizenstein E Narita M Mechanisms of hypergammaglobulinemia and impaired antigen-specific humoral immunity in HIV-1 infection. Blood (2004) 103:2180–6. 10.1182/blood-2003-07-2375 14604962

[145] Preite S Baumjohann D Foglierini M Basso C Ronchi F Fernandez Rodriguez BM Somatic mutations and affinity maturation are impaired by excessive numbers of T follicular helper cells and restored by Treg cells or memory T cells. Eur J Immunol (2015) 45:3010–21. 10.1002/eji.201545920 26332258 PMC5054911

[146] Chowdhury A Del Rio PME Tharp GK Trible RP Amara RR Chahroudi A Decreased T follicular regulatory cell/T follicular helper cell (TFH) in simian immunodeficiency virus-infected Rhesus macaques may contribute to accumulation of TFH in chronic infection. J Immunol (2015) 195:3237–47. 10.4049/jimmunol.1502269 26297764 PMC4575868

[147] Blackburn MJ Zhong-Min M Caccuri F McKinnon K Schifanella L Guan Y Regulatory and helper follicular T cells and antibody avidity to simian immunodeficiency virus glycoprotein 120. J Immunol (2015) 195:3227–36. 10.4049/jimmunol.1402699 26297759 PMC4575875

[148] Miller SM Miles B Guo K Folkvord J Meditz AL McCarter MD Follicular regulatory T cells are highly permissive to R5-tropic HIV-1. J Virol (2017) 91. 10.1128/JVI.00430-17 28615202 PMC5553166

[149] Aandahl EM Michaëlsson J Moretto WJ Hecht FM Nixon DF. Human CD4+ CD25+ regulatory T cells control T-cell responses to human immunodeficiency virus and cytomegalovirus antigens. J Virol (2004) 78:2454–9. 10.1128/JVI.78.5.2454-2459.2004 14963140 PMC369239

[150] Carbonneil C Donkova-Petrini V Aouba A Weiss L Defective dendritic cell function in HIV-infected patients receiving effective highly active antiretroviral therapy: neutralization of IL-10 production and depletion of CD4+CD25+ T cells restore high levels of HIV-specific CD4+ T cell responses induced by dendritic cells generated in the presence of IFN-α. J Immunol (2004) 172:7832–40. 10.4049/jimmunol.172.12.7832 15187167

[151] Kinter AL Hennessey M Bell A Kern S Lin Y Daucher M CD25+CD4+ regulatory T cells from the peripheral blood of asymptomatic HIV-infected individuals regulate CD4+ and CD8+ HIV-specific T cell immune responses in vitro and are associated with favorable clinical markers of disease status. J Exp Med (2004) 200:331–43. 10.1084/jem.20032069 15280419 PMC2211981

[152] Weiss L Donkova-Petrini V Caccavelli L Balbo M Carbonneil C Levy Y. Human immunodeficiency virus-driven expansion of CD4+CD25+ regulatory T cells, which suppress HIV-specific CD4 T-cell responses in HIV-infected patients. Blood (2004) 104:3249–56. 10.1182/blood-2004-01-0365 15271794

[153] Kornfeld C Ploquin MJ Pandrea I Faye A Onanga R Apetrei C Antiinflammatory profiles during primary SIV infection in African green monkeys are associated with protection against AIDS. J Clin Invest (2005) 115:1082–91. 10.1172/JCI23006C1 15761496 PMC1062895

[154] Cecchinato V Tryniszewska E Ma ZM Vaccari M Boasso A Tsai W-P Immune activation driven by CTLA-4 blockade augments viral replication at mucosal sites in simian immunodeficiency virus infection. J Immunol (2008) 180:5439–47. 10.4049/jimmunol.180.8.5439 18390726 PMC2768121

[155] Whitney JB Hill AL Sanisetty S Penaloza-MacMaster P Liu J Shetty M Rapid seeding of the viral reservoir prior to SIV viraemia in rhesus monkeys. Nature (2014) 512:74–7. 10.1038/nature13594 25042999 PMC4126858

[156] Kinter AL Horak R Sion M Riggin L McNally J Lin Y CD25+ regulatory T cells isolated from HIV-infected individuals suppress the cytolytic and nonlytic antiviral activity of HIV-specific CD8+ T cells in vitro. AIDS Res Hum Retroviruses (2007) 23:438–50. 10.1089/aid.2006.0162 17411377

[157] Elahi S Dinges WL Lejarcegui N Laing KJ Collier AC Koelle DM Protective HIV-specific CD8+ T cells evade Treg cell suppression. Nat Med (2011) 17:989–95. 10.1038/nm.2422 21765403 PMC3324980

[158] Jiang Q Zhang L Wang R Jeffrey J Washburn ML Brouwer D FoxP3^+^CD4^+^ regulatory T cells play an important role in acute HIV-1 infection in humanized *Rag2^-/-^*γ*C*^−/−^ mice in vivo. Blood (2008) 112:2858–68. 10.1182/blood-2008-03-145946 18544681 PMC2556621

[159] Angin M Kwon DS Streeck H Wen F King M Rezai A Preserved function of regulatory T cells in chronic HIV-1 infection despite decreased numbers in blood and tissue. J Infect Dis (2012) 205:1495–500. 10.1093/infdis/jis236 22427677 PMC3415814

[160] Moreno-Fernandez ME Zapata W Blackard JT Franchini G Chougnet CA. Human regulatory T cells are targets for human immunodeficiency virus (HIV) infection, and their susceptibility differs depending on the HIV type 1 strain. J Virol (2009) 83:12925–33. 10.1128/JVI.01352-09 19828616 PMC2786841

[161] Pion M Jaramillo-Ruiz D Martinez A Munoz-Fernandez MA Correa-Rocha R. HIV infection of human regulatory T cells downregulates Foxp3 expression by increasing DNMT3b levels and DNA methylation in the FOXP3 gene. AIDS (2013) 27:2019–29. 10.1097/QAD.0b013e32836253fd 24201117

[162] Angin M Sharma S King M Murooka TT Ghebremichael M Mempel TR HIV-1 infection impairs regulatory T-cell suppressive capacity on a per-cell basis. J Infect Dis (2014) 210:899–903. 10.1093/infdis/jiu188 24664171 PMC4192052

[163] Oswald-Richter K Grill SM Shariat N Leelawong M Sundrud MS Haas DW HIV infection of naturally occurring and genetically reprogrammed human regulatory T-cells. PLoS Biol (2004) 2:e198. 10.1371/journal.pbio.0020198 15252446 PMC449855

[164] Tran T-A de Goër de Herve M-G Hendel-Chavez H Dembele B Le Névot E Abbed K Resting regulatory CD4 T cells: a site of HIV persistence in patients on long-term effective antiretroviral therapy. PLoS One (2008) 3:e3305. 10.1371/journal.pone.0003305 18827929 PMC2551739

[165] Jiao Y-M Liu C-E Luo L-J Zhu W-J Zhang T Zhang L-G CD4+CD25+CD127 regulatory cells play multiple roles in maintaining HIV-1 p24 production in patients on long-term treatment: HIV-1 p24-producing cells and suppression of anti-HIV immunity. Int J Infect Dis (2015) 37:42–9. 10.1016/j.ijid.2015.06.008 26095899

[166] Dunay GA Solomatina A Kummer S Hüfner A Bialek JK Eberhard JM Assessment of the HIV-1 reservoir in CD4+ regulatory T cells by a droplet digital PCR based approach. Virus Res (2017) 240:107–11. 10.1016/j.virusres.2017.07.008 28720421

[167] Pandrea I Gaufin T Brenchley JM Gautam R Monjure C Gautam A Cutting edge: experimentally induced immune activation in natural hosts of simian immunodeficiency virus induces significant increases in viral replication and CD4+ T cell depletion. J Immunol (2008) 181:6687–91. 10.4049/jimmunol.181.10.6687 18981083 PMC2695139

[168] He T Brocca-Cofano E Policicchio BB Sivanandham R Gautam R Raehtz KD Cutting edge: T regulatory cell depletion reactivates latent simian immunodeficiency virus (SIV) in controller macaques while boosting SIV-specific T lymphocytes. J Immunol (2016) 197:4535–9. 10.4049/jimmunol.1601539 27837106 PMC5441309

[169] Shirley M. Daclizumab: a review in relapsing multiple sclerosis. Drugs (2017) 77:447–58. 10.1007/s40265-017-0708-2 28211007

[170] Milo R. The efficacy and safety of daclizumab and its potential role in the treatment of multiple sclerosis. Ther Adv Neurol Disord (2014) 7:7–21. 10.1177/1756285613504021 24409199 PMC3886384

[171] Waldmann TA White JD Carrasquillo JA Reynolds JC Paik CH Gansow OA Radioimmunotherapy of interleukin-2R alpha-expressing adult T-cell leukemia with Yttrium-90-labeled anti-Tac. Blood (1995) 86:4063–75. 7492762

[172] Waldmann TA. Daclizumab (anti-Tac, Zenapax) in the treatment of leukemia/lymphoma. Oncogene (2007) 26:3699–703. 10.1038/sj.onc.1210368 17530023

[173] Wang Z Wei M Zhang H Chen H Germana S Huang CA Diphtheria-toxin based anti-human CCR4 immunotoxin for targeting human CCR4(+) cells in vivo. Mol Oncol (2015) 9:1458–70. 10.1016/j.molonc.2015.04.004 25958791 PMC5528803

[174] Wang Z Pratts SG Zhang H Spencer PJ Yu R Tonsho M Treg depletion in non-human primates using a novel diphtheria toxin-based anti-human CCR4 immunotoxin. Mol Oncol (2016) 10:553–65. 10.1016/j.molonc.2015.11.008 26643572 PMC4826841

[175] Ni X Jorgensen JL Goswami M Challagundla P Decker WK Kim YH Reduction of regulatory T cells by Mogamulizumab, a defucosylated anti-CC chemokine receptor 4 antibody, in patients with aggressive/refractory mycosis fungoides and Sezary syndrome. Clin Cancer Res (2015) 21:274–85. 10.1158/1078-0432.CCR-14-0830 25376389

[176] Ogura M Ishida T Hatake K Taniwaki M Ando K Tobinai K Multicenter phase II study of mogamulizumab (KW-0761), a defucosylated anti-cc chemokine receptor 4 antibody, in patients with relapsed peripheral T-cell lymphoma and cutaneous T-cell lymphoma. J Clin Oncol (2014) 32:1157–63. 10.1200/JCO.2013.52.0924 24616310

[177] Walter S Weinschenk T Stenzl A Zdrojowy R Pluzanska A Szczylik C Multipeptide immune response to cancer vaccine IMA901 after single-dose cyclophosphamide associates with longer patient survival. Nat Med (2012) 18:1254–61. 10.1038/nm.2883 22842478

[178] Ghiringhelli F Menard C Puig PE Ladoire S Roux S Martin F Metronomic cyclophosphamide regimen selectively depletes CD4+CD25+ regulatory T cells and restores T and NK effector functions in end stage cancer patients. Cancer Immunol Immunother (2007) 56:641–8. 10.1007/s00262-006-0225-8 16960692 PMC11030569

[179] Wightman F Solomon A Kumar SS Urriola N Gallagher K Hiener B Effect of ipilimumab on the HIV reservoir in an HIV-infected individual with metastatic melanoma. AIDS (2015) 29:504–6. 10.1097/QAD.0000000000000562 25628259 PMC4492799

[180] Hryniewicz A Boasso A Edghill-Smith Y Vaccari M Fuchs D Venzon D CTLA-4 blockade decreases TGF-β, IDO, and viral RNA expression in tissues of SIVmac251-infected macaques. Blood (2006) 108:3834–42. 10.1182/blood-2006-04-010637 16896154 PMC1895471

[181] Boasso A Vaccari M Fuchs D Hardy AW Tsai W-P Tryniszewska E Combined effect of antiretroviral therapy and blockade of IDO in SIV-infected Rhesus macaques. J Immunol (2009) 182:4313–20. 10.4049/jimmunol.0803314 19299731 PMC7249223

[182] Vaccari M Boasso A Fenizia C Fuchs D Hryniewicz A Morgan T Fatal pancreatitis in simian immunodeficiency virus SIVmac251-infected macaques treated with 2′,3′-dideoxyinosine and stavudine following cytotoxic-T-lymphocyte-associated antigen 4 and indoleamine 2,3-dioxygenase blockade. J Virol (2012) 86:108–13. 10.1128/JVI.05609-11 22013040 PMC3255892

[183] Queen C Schneider WP Selick HE Payne PW Landolfi NF Duncan JF A humanized antibody that binds to the interleukin 2 receptor. Proc Natl Acad Sci U S A (1989) 86:10029–33. 10.1073/pnas.86.24.10029 2513570 PMC298637

[184] Williams DP Parker K Bacha P Bishai W Borowski M Genbauffe F Diphtheria toxin receptor binding domain substitution with interleukin-2: genetic construction and properties of a diphtheria toxin-related interleukin-2 fusion protein. Protein Eng (1987) 1:493–8. 10.1093protein/1.6.493 3334101

[185] Collier RJ Diphtheria toxin: mode of action and structure. Bacteriol Rev (1975) 39:54–85. 164179 10.1128/br.39.1.54-85.1975 PMC413884

[186] Kaminetzky D Hymes KB. Denileukin diftitox for the treatment of cutaneous T-cell lymphoma. Biologics (2008) 2:717–24. 10.2147/BTT.S3084 19707452 PMC2727893

[187] Atchison E Eklund J Martone B Wang L Gidron A Macvicar G A pilot study of denileukin diftitox (DD) in combination with high-dose interleukin-2 (IL-2) for patients with metastatic renal cell carcinoma (RCC). J Immunother (2010) 33:716–22. 10.1097/CJI.0b013e3181e4752e 20664355

[188] Foss FM Sjak-Shie N Goy A Jacobsen E Advani R Smith MR A multicenter phase II trial to determine the safety and efficacy of combination therapy with denileukin diftitox and cyclophosphamide, doxorubicin, vincristine and prednisone in untreated peripheral T-cell lymphoma: the CONCEPT study. Leuk Lymphoma (2013) 54:1373–9. 10.3109/10428194.2012.742521 23278639

[189] Telang S Rasku MA Clem AL Carter K Klarer AC Badger WR Phase II trial of the regulatory T cell-depleting agent, denileukin diftitox, in patients with unresectable stage IV melanoma. BMC Cancer (2011) 11:515. 10.1186/1471-2407-11-515 22165955 PMC3293785

[190] Baur AS Lutz MB Schierer S Beltrame L Theiner G Zinser E Denileukin diftitox (ONTAK) induces a tolerogenic phenotype in dendritic cells and stimulates survival of resting Treg. Blood (2013) 122:2185–94. 10.1182/blood-2012-09-456988 23958949

[191] Waldmann TA White JD Goldman CK Top L Grant A Bamford R The interleukin-2 receptor: a target for monoclonal antibody treatment of human T-cell lymphotrophic virus I-induced adult T-cell leukemia. Blood (1993) 82:1701–12. 8400227

[192] O’Mahony D Morris J Carrasquillo J Le N Paik C Whatley M Phase I/II study of Yttrium-90 labeled humanized anti-Tac (HAT) monoclonal antibody and calcium DTPA in CD25-expressing malignancies. J Nucl Med (2006) 47:98.

[193] Vincenti F Kirkman R Light S Bumgardner G Pescovitz M Halloran P Interleukin-2–receptor blockade with daclizumab to prevent acute rejection in renal transplantation. N Engl J Med (1998) 338:161–5. 10.1056/NEJM199801153380304 9428817

[194] Li G Nunoya JI Cheng L Reszka-Blanco N Tsao LC Jeffrey J Regulatory T cells contribute to HIV-1 reservoir persistence in CD4 T cells through cAMP-dependent mechanisms in humanized mice in vivo. J Infect Dis (2017) 216(12):1579–91. 10.1093/infdis/jix547 29045701 PMC5853220

[195] Ma D Xu C Cillo AR Policicchio B Kristoff J Haret-Richter G Simian immunodeficiency virus SIVsab infection of Rhesus macaques as a model of complete immunological suppression with persistent reservoirs of replication-competent virus: implications for cure research. J Virol (2015) 89:6155–60. 10.1128/JVI.00256-15 25833043 PMC4442440

[196] Pandrea I Gaufin T Gautam R Kristoff J Mandell D Montefiori D Functional cure of SIVagm infection in rhesus macaques results in complete recovery of CD4+ T cells and is reverted by CD8+ cell depletion. PLoS Pathog (2011) 7:e1002170. 10.1371/journal.ppat.1002170 21829366 PMC3150280

[197] Peraino JS Zhang H Rajasekera PV Wei M Madsen JC Sachs DH Diphtheria toxin-based bivalent human IL-2 fusion toxin with improved efficacy for targeting human CD25(+) cells. J Immunol Methods (2014) 405:57–66. 10.1016/j.jim.2014.01.008 24462799 PMC4120078

[198] Wang Z Zheng Q Zhang H Bronson RT Madsen JC Sachs DH Ontak-like human IL-2 fusion toxin. J Immunol Methods (2017) 448:51–8. 10.1016/j.jim.2017.05.008 28551309 PMC5576150

[199] Ishida T Ueda R. CCR4 as a novel molecular target for immunotherapy of cancer. Cancer Sci (2006) 97:1139–46. 10.1111/j.1349-7006.2006.00307.x 16952304 PMC11159356

[200] Nishikawa H Sakaguchi S. Regulatory T cells in tumor immunity. Int J Cancer (2010) 127:759–67. 10.1002/ijc.25429 20518016

[201] Iellem A Mariani M Lang R Recalde H Panina-Bordignon P Sinigaglia F Unique chemotactic response profile and specific expression of chemokine receptors CCR4 and CCR8 by CD4(+)CD25(+) regulatory T cells. J Exp Med (2001) 194:847–53. 10.1084/jem.194.6.847 11560999 PMC2195967

[202] Agrawal L Vanhorn-Ali Z Alkhatib G. Multiple determinants are involved in HIV coreceptor use as demonstrated by CCR4/CCL22 interaction in peripheral blood mononuclear cells (PBMCs). J Leukoc Biol (2002) 72:1063–74. 10.1189/jlb.72.5.1063 12429730

[203] Appelbaum FR Sullivan KM Buckner CD Clift RA Deeg HJ Fefer A Treatment of malignant lymphoma in 100 patients with chemotherapy, total body irradiation, and marrow transplantation. J Clin Oncol (1987) 5:1340–7. 10.1200/JCO.1987.5.9.1340 3305793

[204] McCune WJ Golbus J Zeldes W Bohlke P Dunne R Fox DA. Clinical and immunologic effects of monthly administration of intravenous cyclophosphamide in severe systemic lupus erythematosus. N Engl J Med (1988) 318:1423–31. 10.1056/NEJM198806023182203 3259286

[205] Gladstone DE Prestrud AA Pradhan A Styler MJ Topolsky DL Crilley PA High-dose cyclophosphamide for severe systemic lupus erythematosus. Lupus (2002) 11:405–10. 10.1191/0961203302lu229oa 12195780

[206] Petri M Brodsky RA Jones RJ Gladstone D Fillius M Magder LS High dose cyclophosphamide versus monthly intravenous cyclophosphamide for systemic lupus erythematosus. Arthritis Rheum (2010) 62:1487–93. 10.1002/art.27371 20131296 PMC2911961

[207] Glode LM Barqawi A Crighton F Crawford ED Kerbel R. Metronomic therapy with cyclophosphamide and dexamethasone for prostate carcinoma. Cancer (2003) 98:1643–8. 10.1002/cncr.11713 14534880

[208] Lutsiak MEC Semnani RT De Pascalis R Kashmiri SVS Schlom J Sabzevari H Inhibition of CD4^+^25^+^ T regulatory cell function implicated in enhanced immune response by low-dose cyclophosphamide. Blood (2005) 105:2862–8. 10.1182/blood-2004-06-2410 15591121

[209] Ikezawa Y Nakazawa M Tamura C Takahashi K Minami M Ikezawa Z. Cyclophosphamide decreases the number, percentage and the function of CD25+ CD4+ regulatory T cells, which suppress induction of contact hypersensitivity. J Dermatol Sci (2005) 39:105–12. 10.1016/j.jdermsci.2005.02.002 15899580

[210] Heylmann D Bauer M Becker H van Gool S Bacher N Steinbrink K Human CD4+CD25+ regulatory T cells are sensitive to low dose cyclophosphamide: implications for the immune response. PLoS One (2013) 8:e83384. 10.1371/journal.pone.0083384 24376696 PMC3871695

[211] Zhao J Cao Y Lei Z Yang Z Zhang B Huang B. Selective depletion of CD4^+^CD25^+^Foxp3^+^ regulatory T cells by low-dose cyclophosphamide is explained by reduced intracellular ATP levels. Cancer Res (2010) 70:4850–8. 10.1158/0008-5472.CAN-10-0283 20501849

[212] Loyher PL Rochefort J Baudesson de Chanville C Hamon P Lescaille G Bertolus C CCR2 influences T regulatory cell migration to tumors and serves as a biomarker of cyclophosphamide sensitivity. Cancer Res (2016) 76:6483–94. 10.1158/0008-5472.CAN-16-0984 27680685

[213] Nakahara T Uchi H Lesokhin AM Avogadri F Rizzuto GA Hirschhorn-Cymerman D Cyclophosphamide enhances immunity by modulating the balance of dendritic cell subsets in lymphoid organs. Blood (2010) 115:4384–92. 10.1182/blood-2009-11-251231 20154220 PMC2881499

[214] Camisaschi C Filipazzi P Tazzari M Casati C Beretta V Pilla L Effects of cyclophosphamide and IL-2 on regulatory CD4(+) T cell frequency and function in melanoma patients vaccinated with HLA-class I peptides: impact on the antigen-specific T cell response. Cancer Immunol Immunother (2013) 62:897–908. 10.1007/s00262-013-1397-7 23589107 PMC3634989

[215] Alonso CM Lozada CJ. Effects of IV cyclophosphamide on HIV viral replication in a patient with systemic lupus erythematosus. Clin Exp Rheumatol (2000) 18:510–2. 10949730

[216] Bartlett JA Miralles GD Sevin AD Silberman M Pruitt SK Ottinger J Addition of cyclophosphamide to antiretroviral therapy does not diminish the cellular reservoir in HIV-infected persons. AIDS Res Hum Retroviruses (2002) 18:535–43. 10.1089/088922202753747888 12036483

[217] McGary CS Deleage C Harper J Micci L Ribeiro SP Paganini S CTLA-4+PD-1- memory CD4+ T cells critically contribute to viral persistence in antiretroviral therapy-suppressed, SIV-infected Rhesus macaques. Immunity (2017) 47:776–88.e5. 10.1016/j.immuni.2017.09.018 29045906 PMC5679306

[218] Kaufmann DE Kavanagh DG Pereyra F Zaunders JJ Mackey EW Miura T Upregulation of CTLA-4 by HIV-specific CD4+ T cells correlates with disease progression and defines a reversible immune dysfunction. Nat Immunol (2007) 8:1246–54. 10.1038/ni1515 17906628

[219] Elrefaei M Burke CM Baker CAR Jones NG Bousheri S Bangsberg DR HIV-specific TGF-β-positive CD4(+) T cells do not express regulatory surface markers and are regulated by CTLA-4. AIDS Res Hum Retroviruses (2010) 26:329–37. 10.1089/aid.2009.0149 20433405 PMC2933167

[220] Asano T Meguri Y Yoshioka T Kishi Y Iwamoto M Nakamura M PD-1 modulates regulatory T-cell homeostasis during low-dose interleukin-2 therapy. Blood (2017) 129:2186–97. 10.1182/blood-2016-09-741629 28151427 PMC5391624

[221] Wang W Lau R Yu D Zhu W Korman A Weber J PD1 blockade reverses the suppression of melanoma antigen-specific CTL by CD4(+)CD25(Hi) regulatory T cells. Int Immunol (2009) 21:1065–77. 10.1093/intimm/dxp072 19651643 PMC2731790

[222] Streeck H Brumme ZL Anastario M Cohen KW Jolin JS Meier A Antigen load and viral sequence diversification determine the functional profile of HIV-1-specific CD8+ T cells. PLoS Med (2008) 5:e100 10.1371/journal.pmed.0050100 18462013 PMC2365971

[223] Blackburn SD Shin H Haining WN Zou T Workman CJ Polley A Coregulation of CD8+ T cell exhaustion by multiple inhibitory receptors during chronic viral infection. Nat Immunol (2009) 10:29–37. 10.1038/ni.1679 19043418 PMC2605166

[224] Catakovic K Klieser E Neureiter D Geisberger R. T cell exhaustion: from pathophysiological basics to tumor immunotherapy. Cell Commun Signal (2017) 15:1. 10.1186/s12964-016-0160-z 28073373 PMC5225559

[225] Boasso A Herbeuval JP Hardy AW Anderson SA Dolan MJ Fuchs D HIV inhibits CD4+ T-cell proliferation by inducing indoleamine 2,3-dioxygenase in plasmacytoid dendritic cells. Blood (2007) 109:3351–9. 10.1182/blood-2006-07-034785 17158233 PMC1852248

